# High–temporal resolution profiling reveals distinct immune trajectories following the first and second doses of COVID-19 mRNA vaccines

**DOI:** 10.1126/sciadv.abp9961

**Published:** 2022-11-11

**Authors:** Darawan Rinchai, Sara Deola, Gabriele Zoppoli, Basirudeen Syed Ahamed Kabeer, Sara Taleb, Igor Pavlovski, Selma Maacha, Giusy Gentilcore, Mohammed Toufiq, Lisa Mathew, Li Liu, Fazulur Rehaman Vempalli, Ghada Mubarak, Stephan Lorenz, Irene Sivieri, Gabriella Cirmena, Chiara Dentone, Paola Cuccarolo, Daniele Roberto Giacobbe, Federico Baldi, Alberto Garbarino, Benedetta Cigolini, Paolo Cremonesi, Michele Bedognetti, Alberto Ballestrero, Matteo Bassetti, Boris P. Hejblum, Tracy Augustine, Nicholas Van Panhuys, Rodolphe Thiebaut, Ricardo Branco, Tracey Chew, Maryam Shojaei, Kirsty Short, Carl G. Feng, PREDICT-19 Consortium, Susu M. Zughaier, Andrea De Maria, Benjamin Tang, Ali Ait Hssain, Davide Bedognetti, Jean-Charles Grivel, Damien Chaussabel

**Affiliations:** ^1^ Research Branch, Sidra Medicine, PO Box 26999, Doha, Qatar.; ^2^ Laboratory of Human Genetics of Infectious Diseases, The Rockefeller University, New York, NY, USA.; ^3^ IRCCS Ospedale Policlinico San Martino, Genoa, Italy.; ^4^ Department of Internal Medicine and Medical Specialties, University of Genoa, Genoa, Italy.; ^5^ Division of Genomics and Translational Biomedicine, College of Health and Life Sciences, Hamad Bin Khalifa University, Doha, Qatar.; ^6^ Division of Infectious Diseases, Department of Health Sciences, University of Genoa, Genoa, Italy.; ^7^ Department of Experimental and Clinical Medicine, School of Internal Medicine, University of Florence, Florence, Italy.; ^8^ Emergency Department, E.O. Ospedali Galliera, Genova, Italy.; ^9^ Azienda Sanitaria Locale 3 Genovese, Genova, Liguria, Italy.; ^10^ Univ. Bordeaux, Department of Public Health, Inserm U1219 Bordeaux Population Health Research Centre, Inria SISTM, F-33000 Bordeaux, France.; ^11^ Sydney Informatic Hub, The University of Sydney, Sydney, New South Wales, Australia.; ^12^ Nepean Clinical School, The University of Sydney, Sydney, New South Wales, Australia.; ^13^ Westmead Institute for Medical Research, Westmead, New South Wales, Australia.; ^14^ Department of Medicine, Sydney Medical School, Nepean Hospital, The University of Sydney, Sydney, New South Wales, Australia.; ^15^ The University of Queensland, School of Chemistry and Molecular Biosciences, St Lucia, Brisbane, Queensland, Australia.; ^16^ Australian Infectious Diseases Research Centre, The University of Queensland, Brisbane, Queensland, Australia.; ^17^ School of Medical Sciences, Faculty of Medicine and Health, The University of Sydney, Sydney, New South Wales, Australia.; ^18^ Tuberculosis Research Program, Centenary Institute, The University of Sydney, Sydney, New South Wales, Australia.; ^19^ College of Medicine, QU Health, Qatar University, PO Box 2713, Doha, Qatar.; ^20^ Medical Intensive Care Unit, Hamad General Hospital, PO BOX 3050, Doha, Qatar.; ^21^ Weill Cornell Medical College, Doha, Qatar.; ^22^ Computational Sciences Department, The Jackson Laboratory, Farmington, CT, USA.

## Abstract

Knowledge of the mechanisms underpinning the development of protective immunity conferred by mRNA vaccines is fragmentary. Here, we investigated responses to coronavirus disease 2019 (COVID-19) mRNA vaccination via high–temporal resolution blood transcriptome profiling. The first vaccine dose elicited modest interferon and adaptive immune responses, which peaked on days 2 and 5, respectively. The second vaccine dose, in contrast, elicited sharp day 1 interferon, inflammation, and erythroid cell responses, followed by a day 5 plasmablast response. Both post-first and post-second dose interferon signatures were associated with the subsequent development of antibody responses. Yet, we observed distinct interferon response patterns after each of the doses that may reflect quantitative or qualitative differences in interferon induction. Distinct interferon response phenotypes were also observed in patients with COVID-19 and were associated with severity and differences in duration of intensive care. Together, this study also highlights the benefits of adopting high-frequency sampling protocols in profiling vaccine-elicited immune responses.

## Teaser

Blood transcriptome profiling at a high temporal frequency precisely delineates the immune response elicited by mRNA vaccines.

## INTRODUCTION

Coronavirus disease 2019 (COVID-19) vaccines are critical to the ongoing efforts to control the severe acute respiratory syndrome coronavirus 2 (SARS-CoV-2) pandemic. To date, 9 vaccines have received some form of approval for use in humans, and phase 3 trials are ongoing for an additional 11 vaccines (*1*). Notable differences exist among the vaccine products in terms of their design, the levels of protection they confer, and the type, incidence, and severity of adverse events they may elicit. Gaining a comprehensive understanding of the immunological factors underpinning the different responses to various vaccines is a major endeavor. Yet, this knowledge is necessary for guiding timely decisions to modulate vaccination protocols (e.g., the use of different types of vaccines for the first and second vaccine doses). This information may also assist in matching individuals with the growing number of available vaccines based on their demographics, health status, or any other relevant clinical/molecular phenotypes.

Blood transcriptome profiling measures the abundance of transcripts in whole blood on a system-wide scale. It was previously used to comprehensively profile the immune responses elicited by vaccines (*2*, *3*). Notably, this approach identified innate immune signatures arising within hours after administering vaccines (*4*). In a recently published report, Arunachalam *et al.* (*5*) described the blood transcriptome profiles measured following the administration of the BNT162b2 mRNA COVID-19 vaccine. They reported the presence of an interferon signature 1 day after the first dose of vaccine that was no longer detectable on day 7. They further found a more comprehensive interferon/inflammatory signature to be present 1 day after administering the second dose of vaccine. However, the sampling schedule used in this study was relatively sparse. The sample collection time points commonly selected in systems vaccinology studies are based on kinetics established for more conventional vaccines, with sampling at days 1 and 7 often assumed to correspond to the peaks of the innate and adaptive immune responses elicited, for instance, by the influenza or pneumococcal vaccines (*6*). However, the precise kinetics of the immune response elicited by mRNA vaccines remains to be established. In the present study, we endeavored to profile the blood transcriptome of individuals before the administration of the first dose of COVID-19 mRNA vaccines and for the following nine consecutive days. Subjects also collected samples for deep serological profiling at three time points. The same sampling and profiling schedule was repeated to assess the response to the second dose of the vaccine. To achieve this, we have adopted an ultralow-volume sampling procedure for self-collection and RNA preservation of a few drops of blood (50 μl) collected by a fingerstick (*7*).

Together, this work permitted the precise delineation of a well-orchestrated immune response to COVID-19 mRNA vaccines and identified marked differences in the magnitude, timing, and nature of the transcriptional signatures elicited by the first and second doses of the vaccine. Most notably, differences in temporal patterns of responsiveness revealed distinct transcriptional components of the interferon response, which is known to play a key role in controlling SARS-CoV-2 infection (*8*) and was also found here to associate with the subsequent development of the antibody response after vaccination.

## RESULTS

### Study design, implementation, and serological profiling

**Fig. 1.**
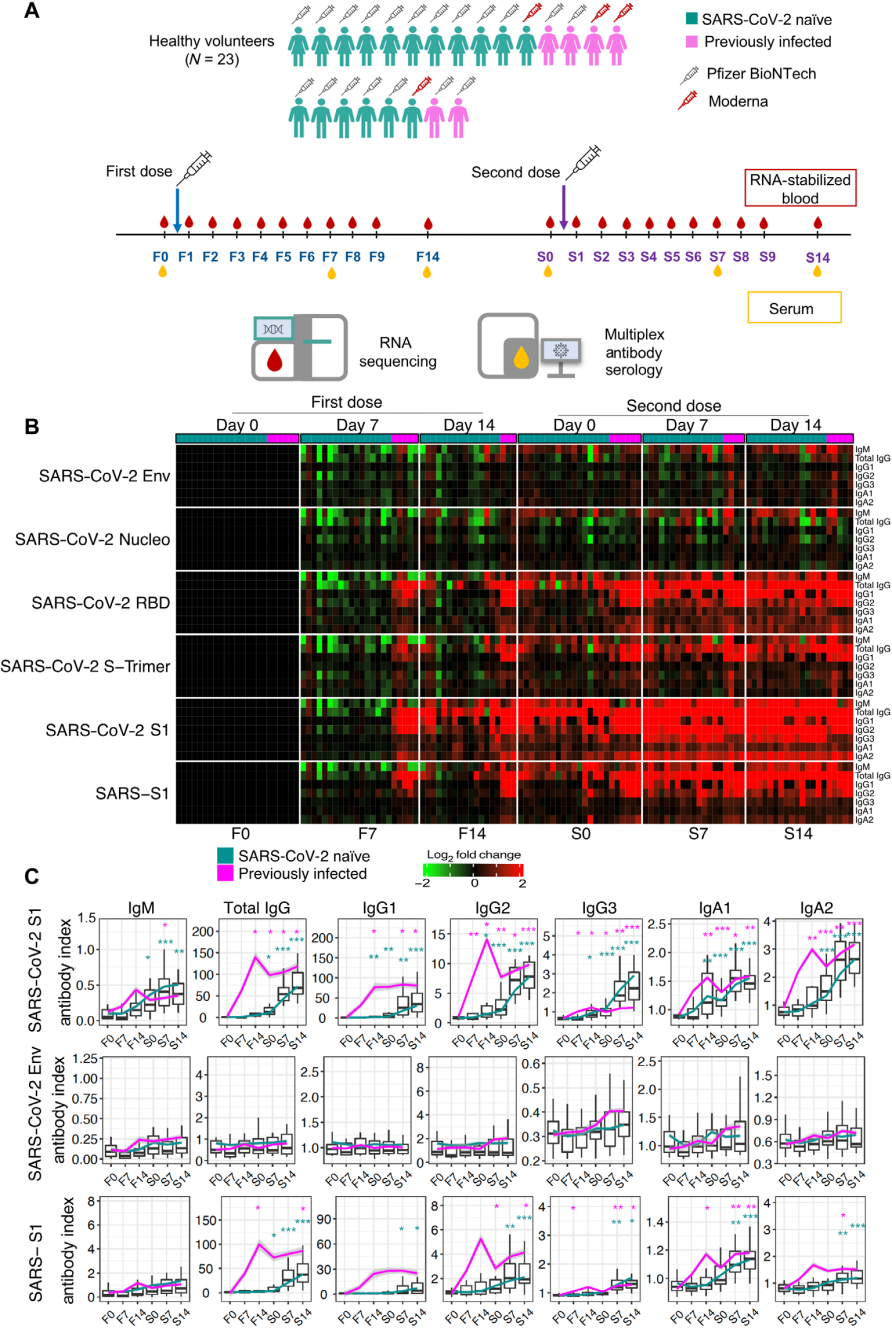
Antibody response to COVID-19 mRNA vaccination. (**A**) Schematic representation of the study design. (**B**) The heatmap represents changes in abundance of antibodies specific to several SARS-CoV-2 antigens and control antigens relative to prevaccination levels. Red indicates a relative increase and green indicates a relative decrease in abundance. Columns represent subjects arranged by time point and have colored tracks at the top indicating whether the subjects were naïve or had previously been infected with SARS-CoV-2. The rows represent antibody reactivities arranged by antigen specificity. (**C**) Changes in antibody levels expressed as an “antibody index” are shown on the box plots, each corresponding to a given antibody type of a given specificity. Lines indicate changes for individuals previously infected with SARS-CoV-2 and who had recovered (in pink) and for naïve individuals (in green). Centerlines, box limits, and whiskers represent the median, interquartile range, and 1.5× interquartile range, respectively. Multiple pairwise tests (paired *t* test) were performed comparing antibody levels to baseline (F0). *P < 0.01, **P < 0.001, and ***P < 0.0001. Tests were run separately for naïve and recovered individuals, as indicated by the colors of the asterisks.

We successfully recruited a cohort of volunteers and implemented a high-frequency sampling protocol (Fig. 1A). This protocol permitted us to ascertain the response to the first and second dose of COVID-19 vaccines at 10 consecutive daily time points: immediately before vaccination and for 9 days after. We collected samples for serological profiling at three time points: before vaccination and on days 7 and 14 after vaccination. We implemented a self-sampling blood collection protocol so that subjects could extract small volumes (50 μl) of RNA-stabilized blood at the required frequency [the approach is described in Materials and Methods and in an earlier publication (*7*)]. We generated RNA sequencing (RNA-seq) profiles using a cost-effective 3′-biased library preparation protocol (Lexogen QuantSeq), which is optimized for optimized for low amounts of RNA input. We generated COVID-19–specific antibody profiles from capillary blood samples collected by volumetric absorptive microsampling and analyzed using a multiplexed bead array established by our team (see Materials and Methods for details). Overall, we enrolled 23 subjects in the study. The characteristics of this cohort are reported in Table 1. They received either two doses of the Pfizer/BioNTech mRNA vaccine (BNT162b2, *N* = 19) or two doses of the Moderna mRNA vaccine (*N* = 4). Among those 23 subjects, 6 had recovered from COVID-19 in the months preceding the administration of the first vaccine dose. In total, we generated 440 RNA-seq profiles and publicly shared this extensive dataset in the Gene Expression Omnibus (GEO) repository under the accession number GSE190001. The serological profiles included reactivity to a stabilized trimer of the Spike protein, its receptor binding domain, the Nucleo and Envelope proteins of SARS-CoV-2, and the subunit S1 of the SARS Spike protein. The data are provided in file S1. We dissected the seroreactivity to each of these antigens by measuring the total immunoglobulin G (IgG), total IgA, and IgM, as well as the finer-scale IgG and IgA subtypes. Serological profiling showed a rise in the levels of antibodies in the subject’s plasma after vaccination (Fig. 1B and fig. S1), including antibodies specific for the SARS-CoV-2 Spike protein, which is targeted by COVID-19 vaccines. No responses to the Envelope protein were detected. Some cross-reactivity was observed with the SARS Spike protein. As expected, higher antibody levels were induced after the first dose in individuals who had been previously infected with the virus (Fig. 1, B and C). These findings are in line with previous reports that have described the serological response to COVID-19 mRNA vaccines (*9*–*12*).

**Table 1.** Subject characteristics. Vaccine type and lot and subjects’ characteristics were recorded, including demographic, biometric data, blood group, underlying diseases, drug usage, and previous COVID-19 disease. Every subject recorded and graded the symptoms that occurred after the first and second vaccination doses, according to the National Institutes of Health “Division of AIDS Acute Events Grading Table.” T2D, type 2 diabetes; NA, not available.

**Patient ID**	**Vaccine name**	**Gender**	**Age**	**Ethnicity**	**Previous COVID-19**	**Underlying disease**	**Drugs**	**Symptoms at first dose (type)**	**Symptoms at first dose (grade)**	**Symptoms at second dose (type)**	**Symptoms at second dose (grade)**
PZB1	Pfizer BioNTech	Female	38	Asian	Yes	No	No	Myalgia	G1	Fever/myalgia	G1
PZB2	Pfizer BioNTech	Male	47	Caucasian	Yes	No	No	Myalgia	G1	Myalgia	G1
PZB3	Pfizer BioNTech	Male	57	Caucasian	Yes	T2D	Metformin, insulin	Myalgia	G1	Myalgia	G1
PZB4	Pfizer BioNTech	Female	34	Indian	No	No	No	None	NA	Chills/insomnia/ headache/myalgia/ fatigue	G3
PZB5	Pfizer BioNTech	Male	38	Indian	No	No	No	Myalgia	G1	Myalgia	G1
PZB6	Pfizer BioNTech	Female	48	Caucasian	No	No	No	Myalgia/ headache	G1	Myalgia	G1
PZB7	Pfizer BioNTech	Female	34	Caucasia/ Arab	No	Hashimoto thyroiditis	No	None	NA	None	NA
PZB8	Pfizer BioNTech	Female	29	Arab	No	No	No	Myalgia/swelling	G1	Fever/myalgia	G1
PZB9	Pfizer BioNTech	Male	41	Arab	No	Allergic rhinitis	No	Myalgia	G1	Myalgia	G1
PZB10	Pfizer BioNTech	Female	35	Arab	No	No	No	Myalgia	G1	Fever/insomnia/ myalgia/fatigue	G2
PZB11	Pfizer BioNTech	Male	41	Caucasian	No	No	No	Myalgia	G2	Myalgia	G1
PZB12	Pfizer BioNTech	Female	34	Indian	No	Hypothyroidism	Levothyroxine	None	NA	Fever/myalgia	G1
PZB13	Pfizer BioNTech	Male	29	Indian	No	No	No	Fatigue	G1	Fever/heaviness in arm	G2
PZB14	Pfizer BioNTech	Female	38	Arab	No	Allergic	No	Myalgia/Headache	G1	Fatigue	G1
PZB15	Pfizer BioNTech	Male	43	Arab	No	Hypothyroidism	Levothyroxine	None	NA	Myalgia/headache	G2
PZB16	Pfizer BioNTech	Female	39	Indian	No	No	No	Heaviness in arm	G1	Fever/myalgia/fatigue	G2
PZB17	Pfizer BioNTech	Female	42	Indian	Yes	T2D, hypertension	Metformin, telmisartan	Fever/headache/ myalgia/fatigue	G2	Fatigue/gastritis	G2
MDA18	Moderna	Male	42	Caucasian	No	Hypertension	Amlodipine, ramipril	Myalgia	G1	Myalgia	G1
PZB19	Pfizer BioNTech	Female	39	Caucasian	No	No	No	Myalgia	G1	Headache/myalgia/ arthralgia	G3
MDA20	Moderna	Female	36	Arab	Yes	No	No	Chills/myalgia	G2	Chills/myalgia	G2
MDA21	Moderna	Female	36	Caucasian	No	No	No	Myalgia	G1	Fever/skin rash/ myalgia	G2
PZB25	Pfizer BioNTech	Female	39	Caucasian	No	No	No	Myalgia	G1	Myalgia	G1
MDA26	Moderna	Female	30	Caucasian	Yes	Asthma	Seretide, salbutamol	Fever/headache/ myalgia/fatigue	G3	Asthma attack/fever/ myalgia/fatigue	G3

Together, the implementation of this protocol established the feasibility of obtaining stabilized RNA blood samples from study subjects after vaccination at high temporal frequencies. We generated a large dataset using a cost-effective RNA-seq protocol that served as the basis for subsequent analyses presented here and was deposited in a public repository. A detailed map of the serological profiles of the subjects enrolled in the study was obtained that permitted us to explore the possible associations between blood transcriptional responses and vaccine immunogenicity.

### The post–first dose interferon response peaks at day 2 and correlates with the antibody response

Innate immune responses are elicited and detectable systemically via blood transcriptome profiling following some but not all vaccination protocols. The aluminum-adjuvanted hepatitis B vaccine is one notable example (*13*). Therefore, our first question was whether transcriptional changes could be observed during the first few days following the administration of COVID-19 mRNA vaccines.

Analyses were carried out using a fixed repertoire of 382 transcriptional modules (BloodGen3) that we have recently established and characterized functionally (see Materials and Methods for details) (*14*). All 23 vaccinated subjects were included in this analysis. Given the small number of subjects who had previously recovered from COVID-19 (*n* = 6), it was not possible to perform separate analyses for the SARS-CoV-2–naïve and SARS-CoV-2–exposed groups. Module responses, corresponding to the percentage of constitutive transcripts for which abundance changes after vaccination, were determined at all time points. The differential gene set enrichment functions of the dearseq R package were run to assess whether changes observed throughout the 9 days after the first dose of vaccination were statistically significant (*15*). This analysis identified significant temporal changes for 22 of the 382 modules constituting the BloodGen3 repertoire (file S2).

**Fig. 2.**
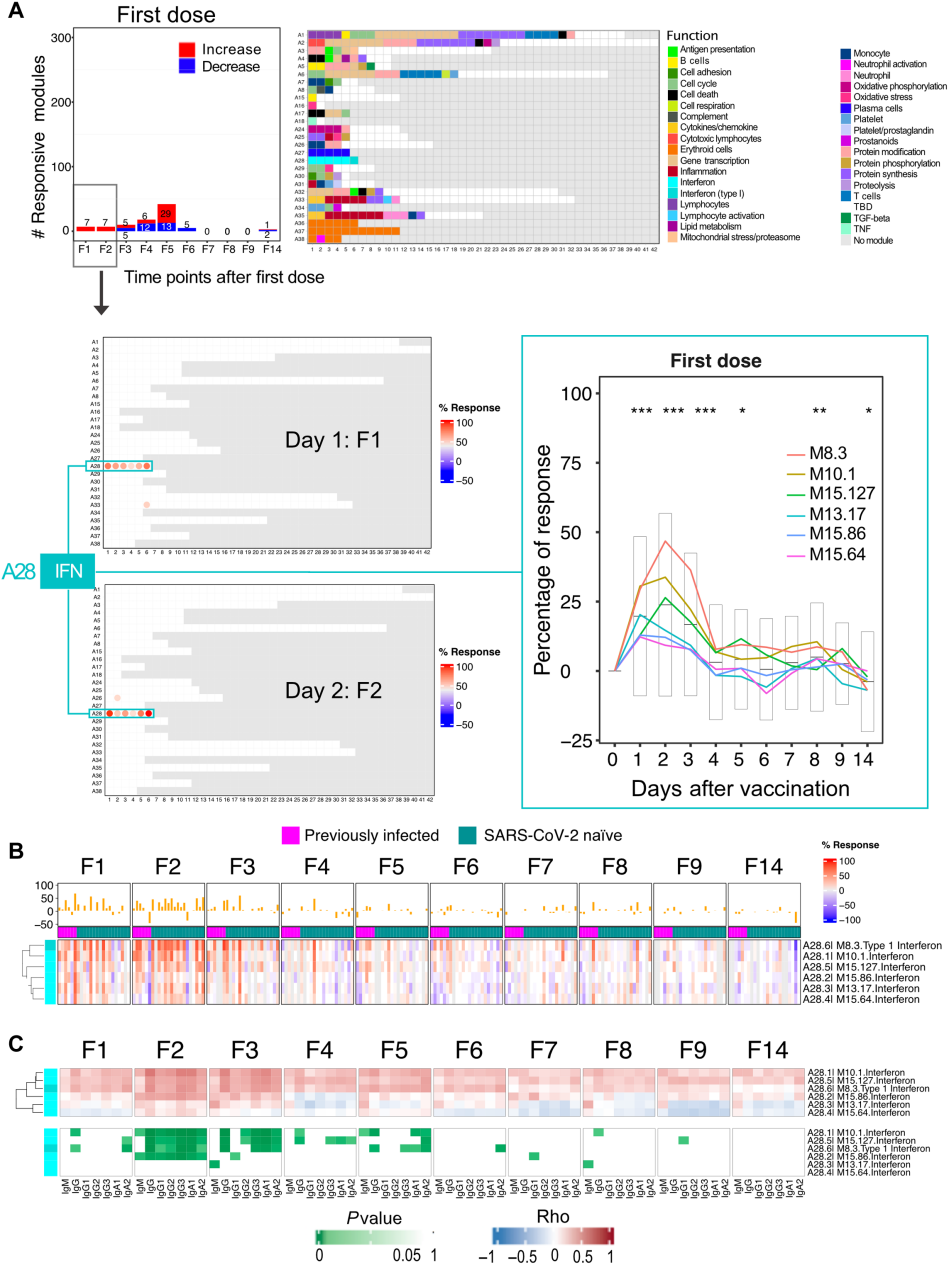
Characterization of the post–first dose interferon response signature. (**A**) The bar graph shows the number of responsive modules (see Materials and Methods) at different days following the administration of the first dose of the vaccine (noted F1 to F14). The fingerprint grid plots represent the overall module responses on days 1 and 2 after the first dose (F1 and F2, respectively). Modules from the BloodGen3 repertoire occupy fixed positions on the fingerprint grids. They are arranged as rows based on membership to module aggregates (rows A1 to A38). Changes compared to the prevaccination baseline are indicated on the grid by red and blue spots of varying color intensity. The color key at the top indicates their assigned function. The line graph shows the average % of responsive transcripts for A28/interferon response modules across all the post–first dose time points. Centerlines, box limits, and whiskers represent the mean, interquartile range, and 1.5× interquartile range, respectively. We also ascertained the significance of changes measured after the first dose at the level of this module aggregate and at each time point (paired *t* test comparing module response at each time point relative to the prevaccination baseline; *P < 0.01, **P < 0.001, and ***P < 0.0001). (**B**) The heatmap represents the proportions of transcripts that changed within the six A28 modules at different time points and across different individuals compared to prevaccination baseline values. Columns represent samples grouped by time point and show profiles of individual subjects within each time point. (**C**) The heatmaps represent associations (Spearman correlation) between levels of module response measured at the prevaccination baseline (F0) and for each of the time points after the first dose (F1 to F9 and F14) and SARS-CoV-2 S1–specific antibody levels measured at the prevaccination baseline (F0) and at 14 days after the first dose (F14). Correlation coefficients are shown at the top (red-white-blue gradient) and respective *P* values are shown at the bottom (white-green gradient).

Only seven modules were found to be changed at any given time point during the first 3 days following the administration of the first dose of the vaccine (Fig. 2A). The abundance of the six modules belonging to the module aggregate A28 appeared to be consistently increased [each “module aggregate” regroups sets of modules that showed consistent abundance profiles across a reference set of 16 disease cohorts that were used for the construction of the BloodGen3 repertoire; see Materials and Methods and (*14*) for details]. The six “aggregate A28” modules are associated with interferon responses (*14*). The gene composition of the modules and the functional annotations are provided here [relevant information is provided in file S3 and can be accessed interactively via https://prezi.com/view/E34MhxE5uKoZLWZ3KXjG/ (*16*)]. The response observed on days 1 and 2 after the first dose of vaccination was mapped onto fingerprint grid plots, where modules occupy a fixed position and are arranged by aggregate. Each aggregate occupies a given row (Fig. 2A). Time-course gene set enrichment analysis indicated that changes observed over the 10 postvaccine time points were significant in four of six A28 modules. The kinetic profiles of the A28 modules showed a peak on day 2 after vaccination. This was also visible on a heatmap showing responses at each time point across individual subjects (Fig. 2B). For each module, the statistical significance of the overall response was determined by time-course gene set enrichment analysis. Four of the six A28 modules met significance thresholds of false discovery rate (FDR) < 0.1 (M8.3: *P* = 1.9 × 10^−4^, FDR = 0.019; M10.1: *P* = 1.9 × 10^−4^, FDR = 0.019; M15.127: *P* = 1.9 × 10^−4^, FDR = 0.019; 727 and M15.86: *P* = 3.9 × 10^−4^, FDR = 0.031) and all six A28 modules, *P* < 0.05 (M13.17: *P* = 1.5 × 10^−3^, FDR = 0.101; M15.64: *P* = 0.044, FDR = 0.727). We next examined whether this signature correlated with antibody responses measured after vaccination. For this, correlation analyses were run at the module level within aggregate A28 using as the endpoint the antibody levels on days 7 and 14 after the first dose and days 7 and 14 after the second dose. “Significance hotspots” are observed when most modules for a given aggregate reach correlation significance thresholds. Figure 2C shows associations between the interferon response measured at multiple time points after the first dose and the antibody response measured on day 14 after the first dose, with fig. S2 showing correlations with day 2 after the first dose (peak interferon response) in individual subjects. Similar comparisons with antibody levels measured at day 7 after the first dose and days 7 and 14 after the second dose are shown in fig. S3. In the case of the post–first dose interferon signature, we identified such significance hotspots on days 2 and 3 for a subset of three interferon modules, M10.1, M15.127, and M8.3, while a fourth module, M15.86, also displayed significant correlations across all antibody types, but only on day 2.

Thus, we found that an interferon response is induced over the first 3 days following the administration of the first dose of mRNA vaccines. Notably, this signature correlated with the antibody response measured 2 weeks later.

### A decrease in inflammation is accompanied by an increase in adaptive immune response genes on day 5 after the first dose

We next characterized the changes occurring beyond the first 3 days following administration of the first vaccine dose. In total, 18 modules displayed changes on day 4 after the first dose, of which 12 showed a decrease in abundance. These modules belonged to three aggregates that have been associated with inflammation (A31, A33, and A35). Most changes were observed on day 4, but for some modules, changes were apparent starting on day 3 and continued beyond day 4, day 5, or even day 6 (fig. S4). In our earlier work, modules within the BloodGen3 aggregate A35 were associated with systemic inflammation mediated by neutrophils and were found to constitute a common denominator across a wide range of pathologies in which systemic inflammation is present (*17*). The association of A35 with inflammatory processes was also ascertained on the basis of functional profiling analysis results and the restriction in expression of its constitutive transcripts observed in multiple reference datasets (*14*). Detailed functional annotations can be accessed via interactive circle packing charts at https://prezi.com/view/7Q20FyW6Hrs5NjMaTUyW/ (*18*). Module aggregate A33 has not been investigated as extensively in any of our prior studies but was clearly associated with inflammation via functional profiling [https://prezi.com/view/VBqKqHuLWCra3OJOIZRR/ (*19*)].

**Fig. 3.**
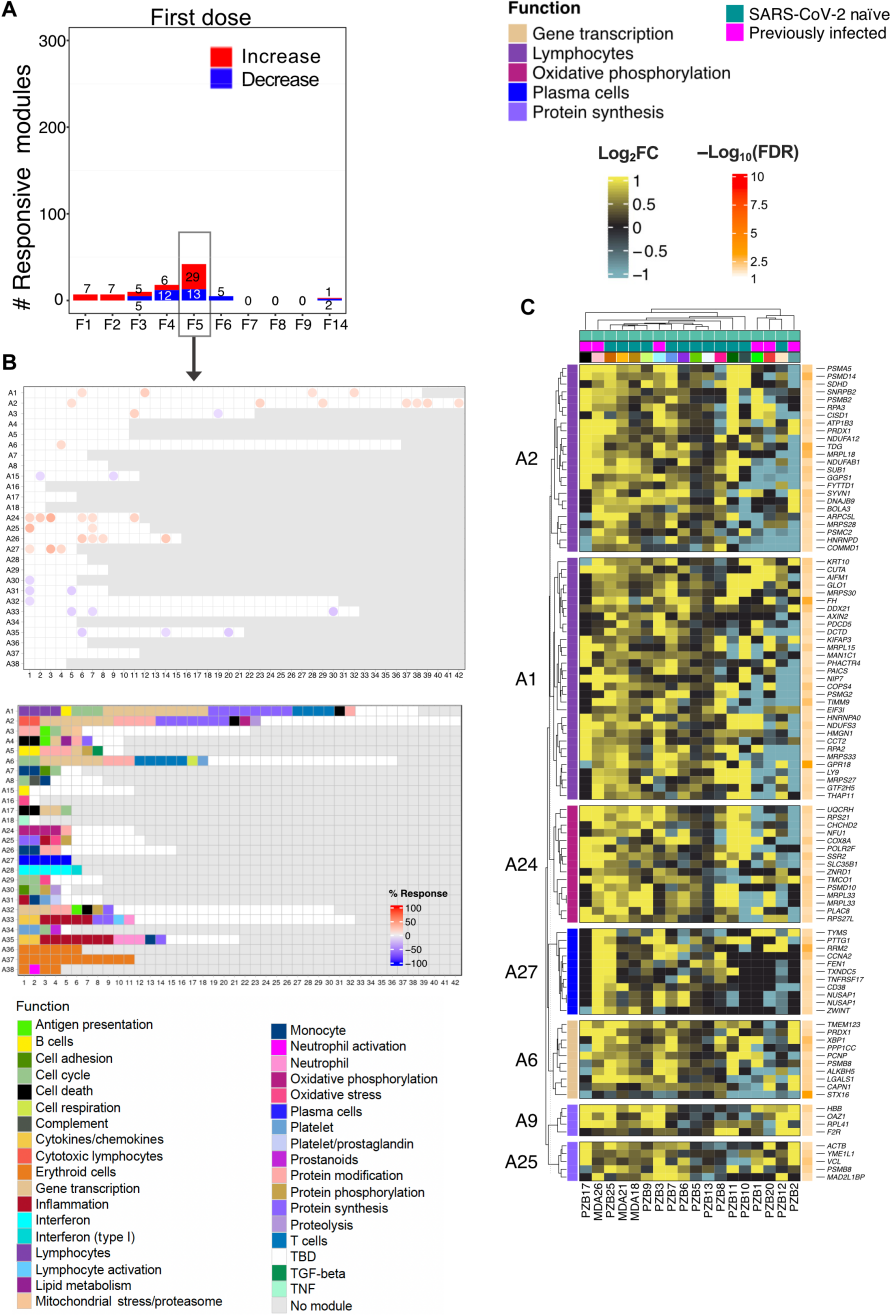
Characterization of responses on day 5 after the first dose. (**A**) The bar graph shows the cumulative number of responsive modules at each time point following the administration of the first dose of the vaccine (noted F1 to F14). (**B**) The fingerprint grid plot shows changes observed at F5 (day 5 after the first dose). The position of the modules on the grid is fixed. The percent response of individual modules is represented on the grid by red and blue spots of varying color intensity denoting a predominant increase or decrease in abundance, respectively. The percentage response of a given module corresponds to the proportion of transcripts predominantly increased or decreased compared to baseline, meeting a significance cutoff of FDR < 0.1. The color key at the top indicates the various functions attributed to the modules that are represented on the grid. (**C**) The heatmap represents log_2_ average FC in abundance of transcripts on day 5 after the first dose (F5). Only modules associated with functional annotations were retained for this figure, and only genes showing significant differences at this time point are shown. Rows represent individual transcripts grouped according to the module aggregate they originate from, corresponding to the different rows on the fingerprint grid plot on the left. Each module aggregate is associated with a unique function, as indicated by the color key above. The columns on the heatmap represent individual subjects coded with the type of vaccine received (at the bottom of the heatmap: Pfizer BioNTech, PZB; Moderna, MDA).

The peak response after the first dose occurred on day 5, with a total of 42 modules showing differences in comparison to the prevaccination baseline (Fig. 3A and fig. S5). At this time point, transcript abundance increased for 29 modules and decreased for 13. Some of the modules that increased at day 5 after vaccination belong to aggregates that are associated with adaptive immune responses. As shown on the fingerprint grid plot presented in Fig. 3B, three of five A27 modules were responsive at this time point. In a reference dataset contributed by Monaco *et al.* (*20*), we found the expression of the genes comprising all five A27 modules to be highly restricted to plasma cells (fig. S6). Notably, the genes comprising one of the A27 modules (M12.15) include the plasmablast marker CD38 and other genes associated with plasmablasts (*IGJ*, *TNFRSF17*, and *TXNDC5*). Detailed annotations and expression profiles of A27 transcripts in the reference datasets can be accessed via https://prezi.com/view/GgIiA0K9kSFHbpVj2I85/ (*21*). The transcriptional profiles of the A27 genes that were significantly changed at this time point are shown on Fig. 3C. However, even for this selection of differentially expressed transcripts, changes were observed only for a subset of subjects at this time point after the first dose, which suggests that the humoral response elicited by the first dose of vaccine is altogether relatively modest. Other immune-relevant modules found to be increased at this time point are associated with T cells [M12.6 from aggregate A1; see https://prezi.com/view/sxap39tKxkmCNTTNIlVO/ (*22*) for functional annotations and Fig. 3C for further details]. Others were mapped to module aggregates A24 and were associated with oxidative phosphorylation, which is known to play a role in T cell activation [6 of 11 modules were responsive; see https://prezi.com/view/eiXvf2LNBLFRgrtaeCuM/ (*23*) for functional annotations and Fig. 3C for further details] (*24*). Other modules have not yet been fully characterized functionally, including, for instance, 4 responsive modules of the 15, belonging to aggregate A26 [see https://prezi.com/view/9CErpW3NwpN2HgRS3Hzf/ (*25*) for functional annotations and Fig. 3C for further details]. Some heterogeneity was observed among study subjects (fig. S5B), most notably with a distinction observed between previously infected individuals, for whom the transcript abundance for those modules tended to decrease (right cluster), while increases were observed in most naïve subjects (left cluster). Notably, the signatures observed on day 5 appeared to be transient, and no modules were increased on day 6 following the administration of the first dose of vaccine.

Together, we found the number of responsive modules to peak on day 5 following the first dose of vaccine. A decrease in the abundance of transcripts associated with inflammation was accompanied by an increase in the abundance of transcripts associated with adaptive immunity. The latter responses appeared to peak earlier than those observed in response to other vaccines, where adaptive response signatures are observed around day 7 after vaccination (*6*, *26*, *27*).

### A marked and polyfunctional response is elicited by the second vaccine dose

**Fig. 4.**
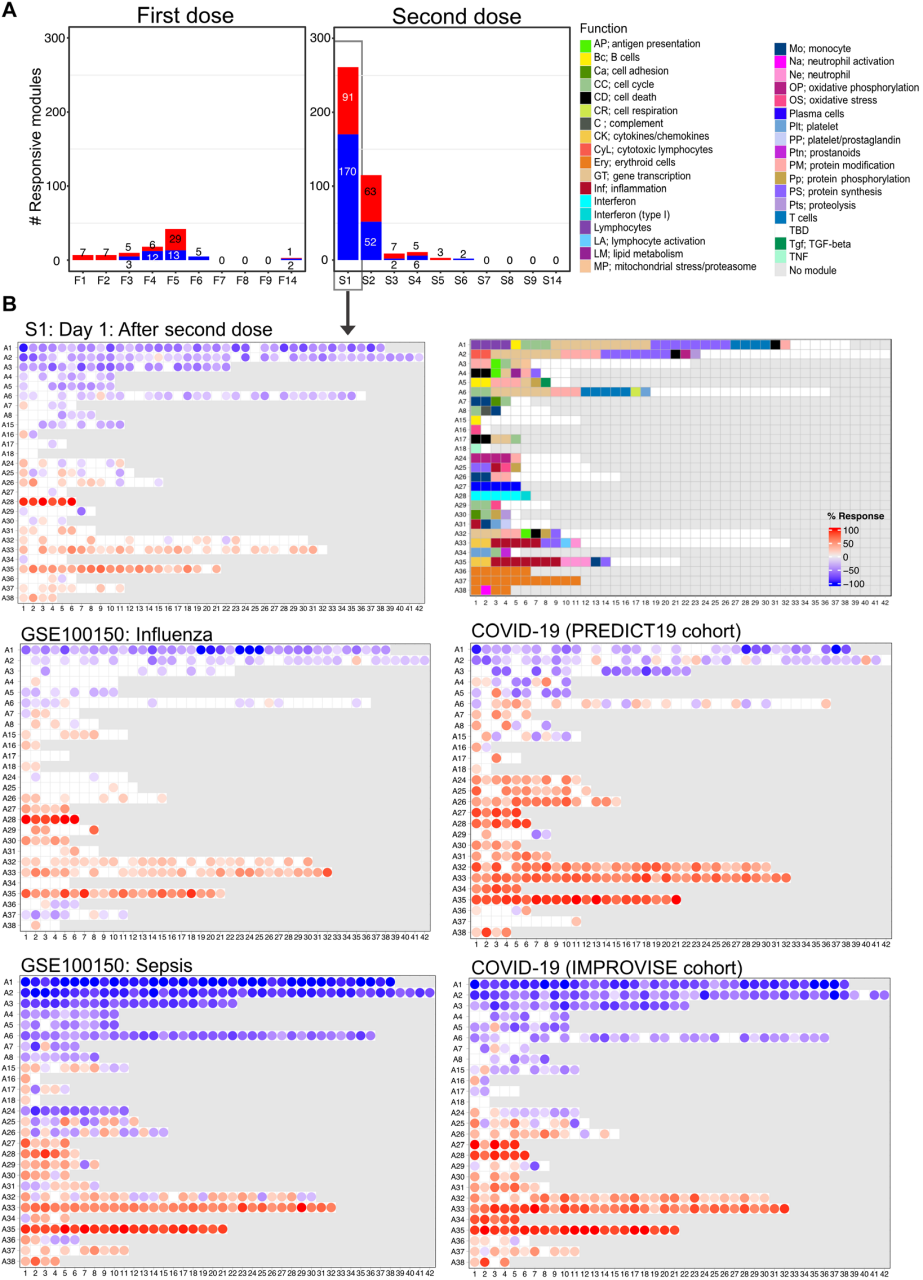
Fingerprint grid plots mapping changes observed on day 1 after the second dose and across reference datasets. (**A**) The bar graphs show the cumulative module response at the various time points after the first and second doses (noted F1 to F14 and S1 to S14, respectively). The *y*-axis values and numbers on the bars indicate the number of modules meeting the 15% response threshold (out of a total of 382 modules constituting the BloodGen3 repertoire, with percentage response corresponding to the proportion of transcripts predominantly increased or decreased compared to baseline, meeting a significance cutoff of DESeq2, FDR < 0.1). (**B**) The fingerprint grid plots show changes in transcript abundance for a given study group in comparison to baseline (prevaccination sample or uninfected control group), with the percent response of individual modules shown by red and blue spots of varying color intensity denoting predominant increase or decrease in abundance, respectively. Changes are shown in the top grid for subjects 1 day after receiving the second dose of COVID-19 mRNA vaccine in comparison with baseline prevaccination samples (this study). Grids in the middle and bottom positions show changes for patients with acute infections caused by influenza virus [earlier work (*14*), with data available in the NCBI GEO repository under accession number GSE100150] or SARS-CoV-2 (this study) and for patients with bacterial sepsis [earlier work (*14*), with data available in the NCBI GEO repository under accession number GSE100150]. The color key at the top indicates the various functions attributed to the modules that occupy a fixed position on the grid plot.

After delineating temporal responses following the administration of the first dose of COVID-19 mRNA vaccine, we examined changes in blood transcript abundance after the second dose. Time-course gene set enrichment analysis identified significant temporal changes for 311 of 382 modules comprising the BloodGen3 repertoire (file S4). After the second dose, the number of responsive modules peaked on day 1, with 261 responsive modules or about two-thirds of the 382 modules constituting the BloodGen3 repertoire (Fig. 4A). This number decreased sharply afterward, with 115 responsive modules on day 2 and only 9 responsive modules on day 3. The kinetic and amplitude of the post–second vaccine dose response contrasted markedly with that observed after the first dose, when, as described above, the number of responsive modules peaked on day 5, with changes found in only 42 modules at that time point.

The day 1 post–second dose response was extensive and polyfunctional (Fig. 4B). An overall decrease in abundance was observed for aggregates broadly associated with lymphocytic cells (aggregates A1 to A8) and increased for module aggregates associated with myeloid cells, inflammation, and circulating erythroid cells (aggregates A33 to A38). In addition, a marked increase in the abundance of modules associated with interferon responses was observed (aggregate A28). We compared the day 1 response fingerprint of the second dose of COVID-19 mRNA vaccine to fingerprints derived from patients with a wide range of pathologies. These included 16 reference datasets encompassing infectious and autoimmune diseases, as well as cancer and solid organ transplant recipients, among others [these cohorts are described in our previously published work (*14*, *28*); the respective blood transcriptome fingerprint collections are accessible via a dedicated web application: https://drinchai.shinyapps.io/BloodGen3Module/]. In addition, we analyzed two original COVID-19 blood transcriptome datasets: One cohort comprised 99 patients with COVID-19 with disease severities ranging from mild and moderate to severe [the “PREDICT-19 (predicting disease progression in severe viral respiratory infections and COVID-19) Consortium Italian cohort dataset”; see Materials and Methods and published study protocol for details (*29*)], while the second cohort comprised 40 patients with COVID-19 recruited at the time of admission to the intensive care unit (ICU) (“IMPROVISE cohort whole blood dataset”). These high-level comparisons showed, first, that the extent of the changes associated with the day 1 response to the second dose of the COVID-19 mRNA vaccine was similar to that observed in some patient cohorts with acute infections (Fig. 4B). More specifically, they were found to most resemble the responses seen in a cohort of subjects with influenza infection, with a marked interferon response (A28) and an inflammation signature (A33 and A35). At a higher level, these response patterns were also generally consistent with those observed in patients with COVID-19 infection. However, the changes that occurred in response to vaccination were not as extreme as those found in patients with sepsis or with the most severe form of COVID-19 (i.e., the IMPROVISE dataset) [most notably for inflammation (A33 and A35) and erythroid cell responses (A36-A38)]. Overall, the BloodGen3 transcriptome fingerprint observed on day 1 after the second vaccine dose contrasted markedly with the fingerprint observed on day 1 after the first.

### A post–second dose interferon signature peaks on day 1 and correlates with antibody response

**Fig. 5.**
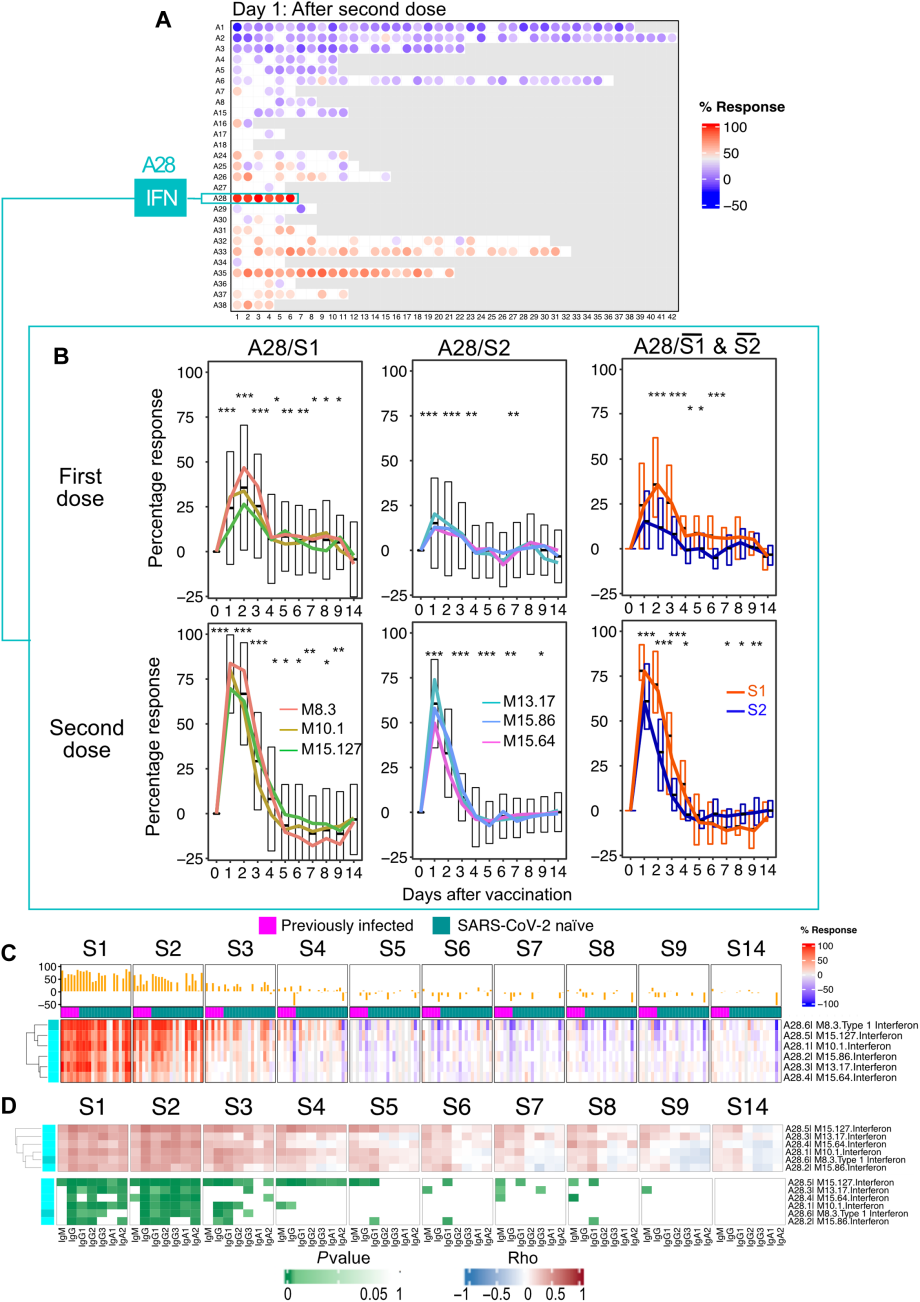
Characterization of the day 1 post–second dose interferon response signature. (**A**) The fingerprint grid plot maps the modular response observed on day 1 after the second dose (% transcripts for a given module showing significant changes, DESeq2, FRD < 0.1). The six modules forming the A28 aggregate are highlighted. (**B**) The line graphs represent the summarized % module responses encompassing all study subjects (one line per module). Changes in abundance are shown after the first dose (top graphs) or after the second dose (bottom graphs) compared to baseline prevaccination levels, for two distinct sets of interferon response modules, A28/S1 and A28/S2 (left and middle, respectively). In addition, the averaged response for A28/S1 and A28/S2 is shown as well (right). Centerlines, box limits, and whiskers represent the mean, interquartile range, and 1.5× interquartile range, respectively. In addition, the significance of changes measured after vaccination (paired *t* test comparing module response at each time point relative to the prevaccination baseline in the graphs on the left and middle) was determined and the averaged A28/S1 and A28/S2 for each subject were compared (*t* test comparing averaged module response for A28/S1 relative to A28/S2 in the graphs on the right). For all tests: *P < 0.01, **P < 0.001, and ***P < 0.0001. (**C**) The heatmap represents the proportions of transcripts that changed within the six A28 modules at different time points and across different individuals compared to prevaccination baseline values. Columns represent samples grouped by time point and show profiles of individual subjects within each time point. (**D**) The heatmaps represent associations (Spearman correlation) between levels of module response measured at the prevaccination baseline (S0) and for each of the time points after the second dose (S1 to S9 and S14) and SARS-CoV-2 S1–specific antibody levels measured at the prevaccination baseline (S0) and at 14 days after the second dose (S14). Correlation coefficients are shown at the top (red-white-blue color gradient) and respective *P* values are shown at the bottom (white-green color gradient).

Despite these marked differences, the interferon response signature was found to be a common denominator between the responses to the first and second doses, since it was observed in both cases in the first few days following administration of the vaccine. We therefore began to dissect the post–second dose response by examining this interferon response signature in more detail (Fig. 5, A and B).

For each module, statistical significance for the overall response was determined by time-course gene set enrichment analysis. Significance was reported after the first dose in Fig. 2. After the second dose, all six A28 modules met significance thresholds of *P* < 0.001 and FDR < 0.001 (M8.3: *P* = 1.9 × 10^−4^, FDR = 3.6 × 10^−4^; M10.1: *P* = 1.9 × 10^−4^, FDR = 3.6 × 10^−4^; M13.17: *P* = 1.9 × 10^−4^, FDR = 3.6 × 10^−4^; M15.127: *P* = 1.9 × 10^−4^, FDR = 3.6 × 10^−4^; M15.64: *P* = 1.9 × 10^−4^, FDR = 3.6 × 10^−4^; M15.86: *P* = 1.9 × 10^−4^, FDR = 3.6 × 10^−4^). We decided to perform hierarchical clustering to identify subsets of modules within the A28 aggregates that might group together on the basis of patterns of transcript abundance across all subjects and time points. Two sets of three modules each were thus identified within the A28 aggregate (Fig. 5B). The first set comprised modules M8.3, M10.1, and M15.127 (referred to as A28/S1), and the second set comprised modules M15.64, M13.17, and M15.86 (referred to as A28/S2). Notably, while modules in A28/S1 peaked on day 2 following the first dose, those belonging to A28/S2 peaked on day 1 (Fig. 5, B and C). Furthermore, A28/S1 modules showed an extended peak after the second dose, with day 2 levels being almost identical to those of the day 1 peak, while A28/S1 modules peaked sharply on day 1, with levels decreasing rapidly thereafter. The fact that these two interferon signatures are indeed distinct was confirmed by comparing aggregated S1 and S2 responses, with statistical differences found across all the early time points following both the first and second doses of vaccines (Fig. 5B, right). This observation was furthermore consistent with our earlier findings in the context of systemic lupus erythematosus (SLE) studies (*30*). We next examined whether transcriptional trajectories for the A28 module aggregate differed between naïve and previously infected individuals (fig. S7A). We found the response to be notably uniform across all subjects after the second dose. Only minor differences were observed between naïve and recovered individuals after the first dose.

Overall, following the administration of the second vaccine dose, the interferon response was noticeably sharper in comparison to the response observed following the first dose and peaked on day 1 instead of day 2. This was illustrated by the difference in the maximum average individual module response across subjects, which, for some of the A28/S1 modules, was close to 50% of the constitutive transcripts on day 2 after the first dose and greater than 80% on day 1 after the second dose. Differences in pattern and amplitude of interferon response between the first and second doses might reflect quantitative and/or qualitative differences in the immune responses being elicited by the vaccine. Publicly available transcriptome profiling data generated before and after interferon treatment in vivo show that A28/S1 modules might be preferentially induced over A28/S2 modules in response to type I interferon (fig. S8) (*31*, *32*), which would be consistent with the pattern of response observed after the first dose. Modules forming the A28/S1 set comprise well-recognized “canonical” interferon response genes, such as oligoadenylate synthetase family members (*OAS1*, *OAS2*, *OAS3*, and *OASL*), interferon-induced protein family members (*IFI6*, *IFI27*, *IFI35*, *IFI44*, and *IFI44L*), and interferon-induced protein with tetratricopeptide repeats family members (*IFIT1*, *IFIT3*, and *IFIT5*) (*14*). Modules forming the A28/S2 set comprise instead most notably members of the nuclear antigen family members *SP100*, *SP110*, and *SP140*, which are associated with interferon-γ signaling, as well as transcription factors *IRF9* and *STAT2*. Composition and functional annotations for A28 modules can be explored further at https://prezi.com/view/E34MhxE5uKoZLWZ3KXjG/ (*16*).

Last, we observed a strong association between the post–second dose interferon signature and the subsequent development of an antibody response (Fig. 5D and figs. S9 to S11). Positive correlations were observed for all six A28 modules that reached significance on days 1, 2, and 3 after the second dose. Notably, this differed from the interferon response observed after the first dose, for which significance was reached only for four of the six modules and only on days 2 and 3.

Together, the resolution of immune trajectories after vaccination via high–temporal frequency profiling permitted the delineation of distinct patterns of interferon responses following the first and second doses of vaccine. One of those module sets, A28/S1, dominated the response elicited by the first dose of vaccine and peaked on day 2. The response following the second dose showed a potent induction of both S1 and S2 and peaked instead on day 1.

### Inflammation and erythroid cell signatures peak sharply on day 1 after the second dose

**Fig. 6.**
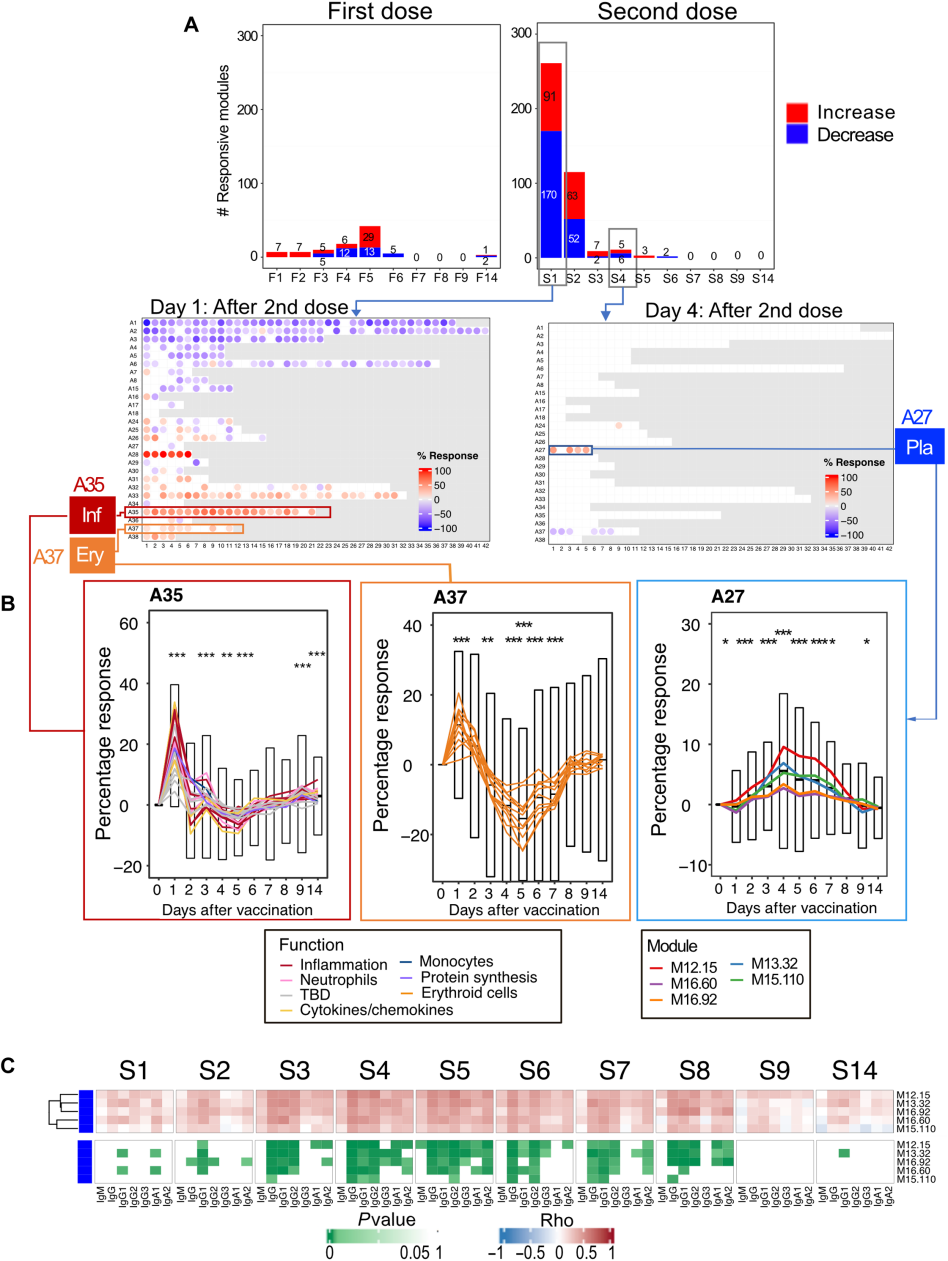
Characterization of post–second dose inflammation, erythroid cell, and plasmablast responses. (**A**) The bar graph at the top represents the number of responsive modules at any given time point after the first and second doses. The fingerprint grid plots below map the modular response observed on day 1 after the second dose (left) and day 4 after the second dose (right). (**B**) The line graphs show the average percentage responses of A35, A37, and A27 modules across multiple time points (left, middle, and right, respectively). Each line represents the profile of the modules constituting a given aggregate. For all line graphs, centerlines, box limits, and whiskers represent the mean, interquartile range, and 1.5× interquartile range, respectively. The significance of changes measured after the second dose was determined at each time point and is shown on the graphs (paired *t* test comparing module response at each time point relative to the prevaccination baseline; *P < 0.01, **P < 0.001, and ***P < 0.0001). (**C**) The heatmaps represent associations between levels of module response measured at the prevaccination baseline (S0) and for each of the time points after the second dose (S1 to S9 and S14) and SARS-CoV-2 S1–specific antibody levels measured at the prevaccination baseline (S0) and at 14 days after the second dose (S14). Specifically, the heatmap at the top (blue-red color gradient) represents the correlation coefficients across multiple days and for each day across multiple subjects, with rows corresponding to the five A27 plasmablast modules. The heatmap below (green color gradient) represents the significance of the correlations shown on the heatmap at the top, with the same order of rows and columns.

We continued the dissection of the day 1 post–second dose signature, focusing this time on responses associated with inflammation and circulating erythroid cell precursors (Fig. 6A). For each module, statistical significance for the overall response was determined by time-course gene set enrichment analysis (see Materials and Methods). For A35, 20 of 21 modules met significance thresholds (*P* < 0.05 and FDR < 0.01). As described earlier, the abundance of A33 and A35 transcripts was decreased on days 4 through 6 following administration of the first dose of vaccine. However, following the second dose, a sharp and transient increase in abundance of the transcript forming these modules was detected instead. A well-delineated response peak was observed on day 1 after the first dose for both the A33 and A35 modules (Fig. 6B, A35, left), but in contrast to the interferon response (A28/S1), it did not extend beyond the first day. Responses in naïve and recovered individuals after the second dose appeared to be similar (fig. S7B). Yet, as shown in the same figure, the decrease in abundance characteristic of the first dose response for this module aggregate tended to be more pronounced in recovered individuals, although it is worth pointing out that this observation is only based on a limited number of individuals (as a reminder, only 6 of the 23 subjects were previously infected and had recovered from COVID-19). As mentioned above, the increase in abundance of A35 modules was recently shown to be a hallmark of the blood transcriptional signature of psoriasis (*17*). This signature was a common denominator across autoimmune and inflammatory diseases and was thought to be driven by neutrophil activation.

Time-course gene set enrichment analysis determined that 11 of 11 modules forming aggregate A37 changed significantly after the second vaccine dose. Modules comprised in the module aggregate A37, which we previously associated with glycophorin A–positive circulating erythroid cell signatures (*28*), also displayed a sharp but transient increase in transcript abundance on day 1 after the second dose (Fig. 6B, A37, middle). However, the abundance tended to dip afterward, with a low peak observed on day 5 after the second dose, before recovering by day 7. To our knowledge, such a circulating erythroid cell signature has not been previously described after vaccination. Last, we did not find evidence of an association between the day 1 post–second vaccine dose, inflammation or erythroid cell signatures, and the antibody responses. Overall, similar trends were observed in naïve and recovered subjects (fig. S7C).

Overall, these findings highlight the marked differences in the kinetics and nature of the immune responses elicited in the first days following the administration of the first and second doses of COVID-19 mRNA vaccines. They also point to the possible “training” of the innate immune response by the first vaccine dose.

### A plasmablast signature peaks on day 4 after administration of the second vaccine dose and correlates with antibody responses

After the second dose of COVID-19 mRNA vaccine, the number of responsive modules peaked sharply on day 1 and then rapidly subsided beyond day 2, with the number of responsive modules on days 3, 4, 5, and 6 being reduced to 9, 11, 3, and 2, respectively. Yet, changes within this later time frame are meaningful, as they specifically concern the set of five modules comprising aggregate A27, which is associated with the presence of antibody-producing cells in the peripheral blood (Fig. 6A).

Three of the five A27 modules showed significant alterations after the second dose (M12.15, M13.32, and M15.110) (Fig. 6B, A27, right). The proportion of differentially expressed transcripts in each module was relatively modest (with an average of 15% at the peak of response), especially in comparison with the interferon signatures described above (with an average of >80% for some modules at the response peak). We also examined the association of this post–second dose plasmablast signature with the antibody response and found a significant association starting from about day 3 and lasting until day 7 after the second dose (Fig. 6C and figs. S12 and S13). Robust responses were observed in both naïve and recovered subjects after the second dose (fig. S7D). However, notably, the A27/plasmablast response was only observed in recovered individuals after the first dose.

In summary, COVID-19 mRNA vaccination induced a marked plasmablast response that peaked on day 4 after vaccination. This was unexpected since such signatures typically are measured around day 7 after vaccine administration [e.g., in the case of influenza or pneumococcal vaccines (*6*)]. We were also able to demonstrate an association between this post–second dose plasmablast signature and the subsequent development of humoral immunity.

### Patterns of interferon induction elicited by COVID-19 mRNA vaccines are also observed among patients with COVID-19

This work has identified the interferon response as the most upstream factor associated with the development of humoral immunity following COVID-19 mRNA vaccination. High–temporal resolution profiling delineated distinct patterns of interferon induction after the first dose and after the second dose, and we next decided to determine whether similar response patterns could be identified among patients with COVID-19 disease.

**Fig. 7.**
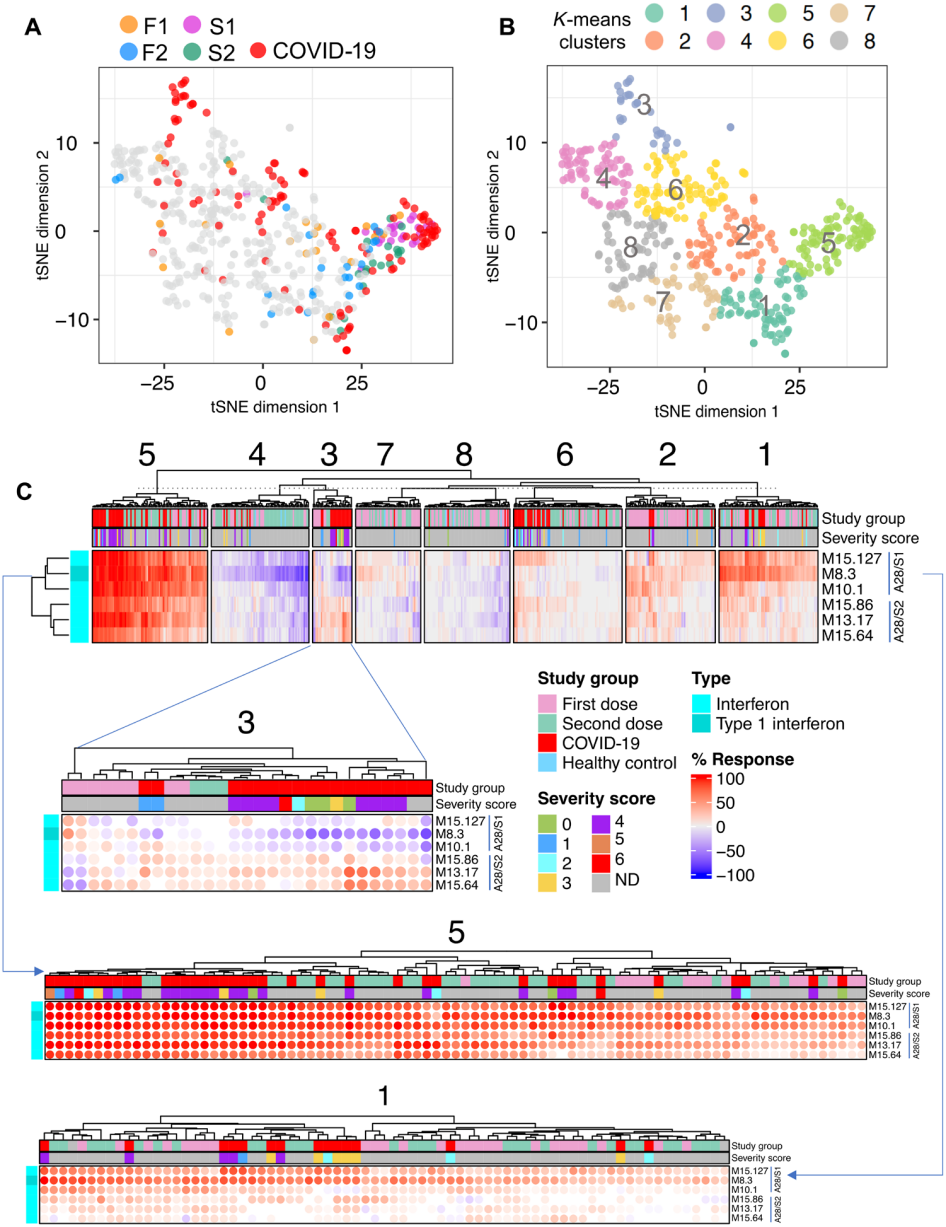
Comparing patterns of interferon response in vaccinated individuals and a cohort of patients with COVID-19. (**A**) The tSNE plot represents similarities in patterns of interferon response induction across the six modules forming aggregate A28 and among samples comprised in our vaccination cohort and one of our COVID-19 disease cohorts (PREDICT-19/Italy). COVID-19 samples are shown in red along with specific postvaccination time points [post–first dose days 1 and 2 (F1 and F2) and post–second dose days 1 and 2 (S1 and S2)]. (**B**) Samples from the consolidated cohorts were partitioned into eight clusters via *k*-means clustering, the distribution of which is shown on this tSNE plot. (**C**) Heatmaps show patterns of response for the six interferon response modules across the eight sample clusters. The red colors indicate that the abundance of transcripts for a given module is predominantly increased with the intensity representing the proportion of constitutive transcripts meeting a given threshold, which, at the level of individual samples, is a fixed FC and difference cutoff (|FC| > 1.5 and |difference| > 10 in a given sample over its respective prevaccination baseline). The blue color denotes a predominant decrease in abundance of constitutive transcripts compared to the same individual’s prevaccination baseline. Details are shown below for clusters 3, 5, and 8 in separate heatmaps.

For this, we relied on blood transcriptome data generated de novo from the PREDICT-19 Consortium Italian COVID-19 cohort comprising 99 patients with a wide spectrum of disease severity. We used the response values for the six interferon modules from aggregate A28 to map individual COVID-19 patient samples along with postvaccine samples on the same t-distributed Stochastic Neighbor Embedding (tSNE) plot (Fig. 7A). First, we confirmed that there was no apparent separation of the vaccination and COVID-19 patient cohorts and that batch correction was therefore not warranted before proceeding with comparative analyses (fig. S14). This is consistent with the results of meta-analyses that we have previously conducted at the module level (*28*). To help with the interpretation, *k*-means clustering was performed using the consolidated set of samples, resulting in the formation of eight distinct clusters. Next, we examined the distribution of samples from the vaccine and COVID-19 cohorts across the tSNE plot and among the eight clusters (Fig. 7B). Time points at which an interferon response was detectable in vaccinated subjects were of particular interest. Day 1 and day 2 post–first dose samples (F1 and F2), while preferentially found in clusters 1 and 5, appeared to be distributed across the entire tSNE plot. This is in contrast with day 1 and day 2 post–second vaccine dose samples (S1 and S2), which were almost exclusively found in cluster 5. A set of patients with COVID-19 also colocalized in cluster 5, while others were found scattered across clusters, especially clusters 1, 2, 6, and 3. Interferon responses were detectable in all these clusters but with important nuances. For one, samples from cluster 5 showed, by far, the most potent responses, with responses seen in most cases across all six interferon modules, which was consistent with the response observed following administration of the second dose of vaccine (Fig. 7C). In comparison, the response was less pronounced in samples from cluster 1, which was dominated by modules associated with type I interferon responses (the A28/S1 set comprising M10.1, M8.3, and M15.127 described above). This was more consistent with the pattern of response observed after the first dose of vaccine. Signatures for samples forming clusters 2 and 6 were not well defined and were, in some cases, absent, yet these clusters also included patients with COVID-19. Samples forming cluster 3 displayed a peculiar signature, with an increase in the abundance of modules belonging to the A28/S2 set (M15.64, M13.17, and M15.86) concomitantly with a decrease in modules forming the A28/S1 set. Among the samples forming this cluster, this pattern was most apparent for the patients with COVID-19.

Thus, we used here the distinct interferon response “traits” observed after COVID-19 vaccination as a benchmark for the interpretation of COVID-19 patient signature. We were able to establish that most patients with COVID-19 display interferon responses consistent with those found after vaccination, which, as established in this study, were associated with the development of potent humoral responses. However, a subset of patients displayed patterns of interferon response that are not typically seen in vaccinated individuals.

### The distinct interferon response signature observed in patients with COVID-19 is associated with a worse course of disease

**Fig. 8.**
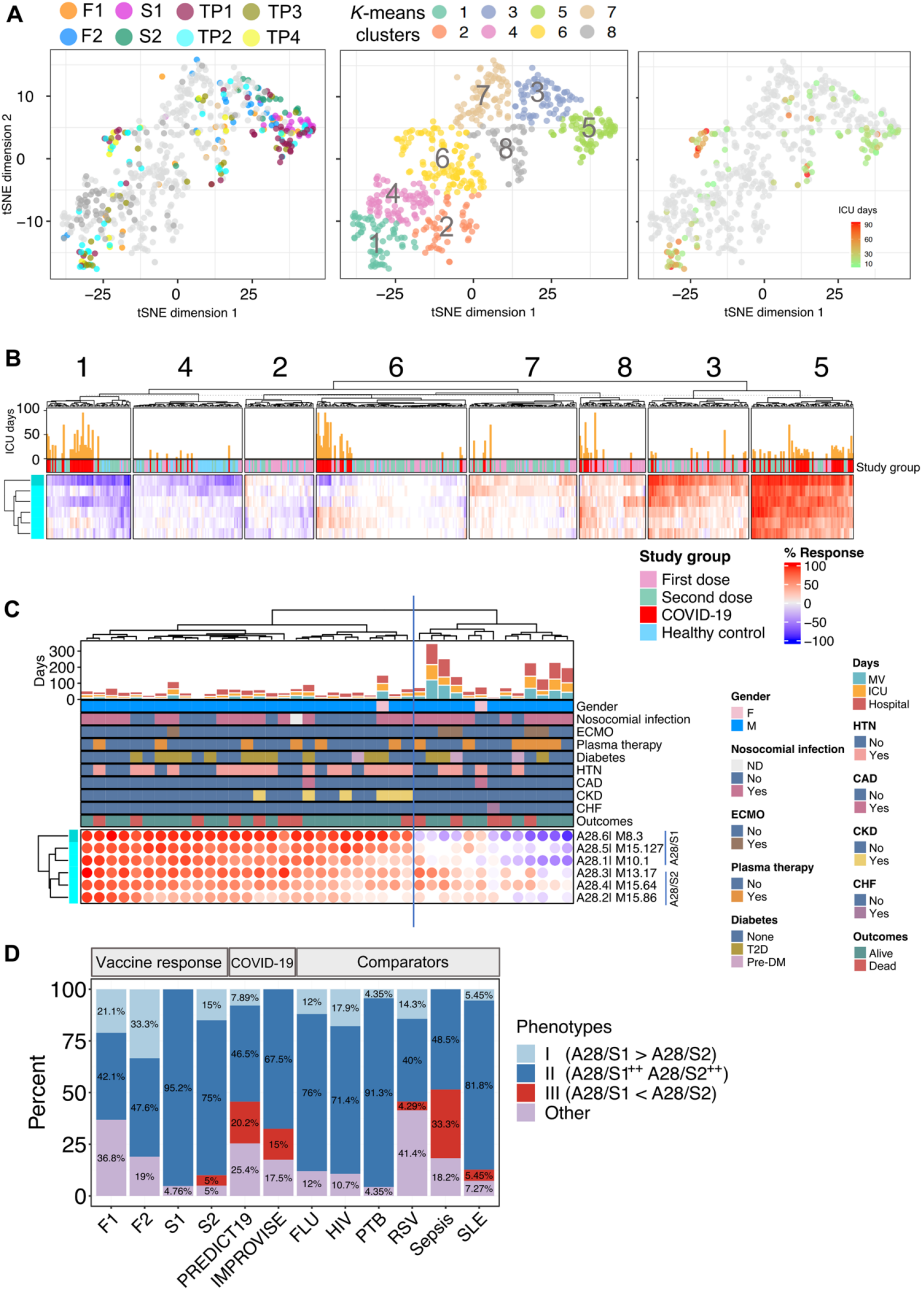
Comparison of interferon response patterns of vaccinated individuals and a cohort of patients with COVID-19 with severe disease under intensive care. (**A**) The tSNE plot represents similarities in patterns of interferon response induction among vaccinated subjects and subjects with COVID-19 (IMPROVISE cohort). Specific postvaccination time points (F1, F2, S1, and S2) and repeat samples from a patient with COVID-19 (TP1 to TP4) are shown. Samples from the consolidated cohorts were partitioned into eight clusters via *k*-means clustering (center). Length of ICU stay is shown on the right. (**B**) The fingerprint heatmap shows patterns of response for the six interferon response modules across the eight sample clusters defined by *k*-means clustering in (A). (**C**) The heatmap shows patterns of interferon responses for patients with COVID-19 upon ICU admission. Multiple clinical parameters are shown on the tracks above [extracorporeal membrane oxygenation (ECMO), hypertension (HTN), coronary artery disease (CAD), chronic kidney disease (CKD), and congestive heart failure (CHF)]. The histogram represents the length of stay in the hospital, in the ICU, and under mechanical ventilation (MV), in days. DM, diabetes mellitus. (**D**) The bar graph represents for different datasets the proportion of samples corresponding to IRTP I, II, or III, according to the following definition, which is based on the delineation of two distinct sets of interferon response modules: A28/S1 (M8.3, M15.127, M10.1) and A28/S2 (M13.17, M15.64, M15.86): IRTP I = (“S1^++^S2^+^,” “S1^++^S2^0^,” and “S1^+^S2^0^”); IRTP II = (S1^++^S2^++^); IRTP III = (“S1-S2^++^,” “S1-S2^+^,” “S1^0^S2^++^”, and “S1^0^S2^+^”). The datasets shown along the *x* axis include day 1 and day 2 post–first and post–second vaccine dose responses (F1, F2, S1, and S2, respectively; *N* = 23); the PREDICT-19 (*N* = 99 patients) and IMPROVISE (*N* = 40 patients) COVID-19 cohorts. Other datasets were derived from an earlier study (*14*) and include reference cohorts of patients with acute influenza infection (FLU; *N* = 25), HIV infection (*N* = 28), active pulmonary tuberculosis (PTB; *N* = 23), acute RSV infection (*N* = 70), bacterial sepsis (*N* = 33), and SLE (*N* = 55).

The fact that some patients with COVID-19 failed to display robust “postvaccine-like” interferon responses may be due to either a defective innate immune response, which may lead to a more severe disease course, or conversely to activation thresholds not being reached in patients presented with milder disease. Thus, we next examined patterns of interferon response in another original COVID-19 disease cohort, composed exclusively of patients enrolled at the time of admission in the ICU (the IMPROVISE cohort, which was also described above). As described above, we mapped individual COVID-19 patient samples along with postvaccine samples on a tSNE plot based on similarities in the patterns of interferon responsiveness across the six A28 interferon modules (Fig. 8A). As was the case earlier for the PREDICT-19 cohort, COVID-19 subjects were found to be distributed throughout multiple clusters. Notably, patients who colocalized with day 1 post–second dose samples tended to have relatively short ICU stays (in cluster 5 with potent A28/S1 and A28/S2 responses), and only a few patients colocalized with day 2 post–first dose samples in cluster 3, which was characterized by a more prominent A28/S1 signature compared with A28/S2 (Fig. 8B). Furthermore, distinct groups of patients in clusters 1 and 6 displayed the peculiar pattern of interferon response dominated by A28/S2 that was identified earlier among patients enrolled in the PREDICT-19 cohort. Notably, patients from the IMPROVISE cohort displaying this pattern of interferon response showed significantly lengthier stays in the ICU compared to patients displaying patterns of interferon response that are consistent with those observed after vaccination (Fig. 8C comparing left and right clusters: for length of hospital stay, *t* test, **P = 0.006; mechanical ventilation days, *P = 0.016; and ICU stay, *P = 0.012; notably, age did not appear to be a factor driving differences in patterns of interferon response and length of ICU stays; fig. S15).

Thus, in a cohort of subjects uniformly presenting with severe disease, post–first dose–like patterns of interferon response dominated by A28/S1 were less prevalent. Post–second dose–like patterns of interferon response characterized by robust A28/S1 and A28/S2 signatures were observed instead in most patients. A notable exception was patients presenting with patterns of response dominated by A28/S2, not observed previously following vaccination but which were found again in this second independent COVID-19 dataset. In this context, we could also establish that such response is associated with a worse disease course. This overall supports the notion that patients harboring this signature may fail to mount an effective immune response against SARS-CoV-2.

### The distinct interferon response phenotype observed in patients with COVID-19 is not typically found in the context of other infections

Last, we asked whether the A28/S2-dominated interferon response pattern associated with worse disease outcomes in patients with COVID-19 was also commonly found in other infectious diseases. For this, we first developed a standard definition of “interferon response transcriptional phenotypes” (IRTPs): The two distinct signatures described above, A28/S1 and A28/S2, were used as traits for the definition of three main phenotypes observed following vaccination and in response to SARS-CoV-2 infection. (i) IRTP I encompassed A28/S1-dominated patterns of response: “A28/S1^++^A28/S2^+^,” “A28/S1^++^A28/S2^0^,” and “A28/S1^+^A28/S2^0^” (see Materials and Methods for details). (ii) IRTP II corresponded to a pattern of interferon response characterized by the strong induction of both components: A28/S1^++^A28/S2^++^. (iii) IRTP III encompassed the A28/S2-dominated patterns of interferon response: “A28/S1^−^A28/S2^++^,” “A28/S1^−^A28/S2^+^,” “A28/S1^0^A28/S2^++^,” and “A28/S1^0^A28/S2^+^.” These three IRTPs were, in turn, used for the stratification of our vaccination cohort at early time points following administration of the first and second vaccine doses, as well as both of our COVID-19 cohorts and of several reference cohorts of patients that we had generated as part of one of our earlier studies (*14*), focusing more particularly on pathologies known to elicit robust interferon responses, including viral infections [influenza, Rous sarcoma virus (RSV), and HIV], tuberculosis, or SLE (Fig. 8D).

IRTP I was found in approximately one-third of the vaccinated subjects at peak response on day 2 after the first dose (Fig. 8D, F2). It was, however, absent at peak response after the second dose (S1). Similarly, IRTP I was found among patients with COVID-19 belonging to the PREDICT-19 cohort (although in only 7.9% of patients), but not among those belonging to the IMPROVISE cohort, who presented with more severe disease. IRTP I was otherwise also found in between 0 and 18% of subjects across most of our reference cohorts. However, as was the case of our severe COVID-19 cohort, it was absent in the comparator cohort of patients with bacterial sepsis. In the context of mRNA vaccination, IRTP II, which is characterized by the robust induction of both A28/S1 and A28/S2 components, was observed following the second dose of vaccines in 95% of samples profiled on day 1, which corresponds to the peak response. The first dose of COVID-19 mRNA vaccine was able to induce both components robustly but in only 48% of samples at peak (day 2 after the first dose). IRTP II was otherwise also prevalent in patients with COVID-19, which is consistent with our earlier observation. It was also found in most samples in the other pathologies used as comparators, except for RSV and bacterial sepsis (40 and 48%, respectively). IRTP III, which is characterized by an A28/S2-dominated response, was observed only rarely after COVID-19 mRNA vaccination. It was, however, prevalent among patients with COVID-19, with 20.2 and 15% of subjects with this phenotype in the PREDICT-19 and IMPROVISE cohorts, respectively. However, it was not observed in patients with tuberculosis, influenza virus, or HIV infection. IRTP III is, on the other hand, found in 4.3% of patients with RSV infection and reached its peak prevalence in patients with bacterial sepsis (33.3%).

In summary, those results show that in most instances, both components of the transcriptional interferon response can be robustly induced following COVID-19 vaccination or viral infection (i.e., corresponding to IRTP II). However, incomplete patterns of induction can also be observed in some circumstances. We hypothesize that this may be due to (i) activation thresholds not being reached, in the case of IRTP I, or (ii) subjects failing to mount an effective interferon response, in the case of IRTP III, which, in the context of SARS-CoV-2 infection, might affect their ability to control the infection. Notably, besides COVID-19, IRTP III phenotypes were only observed in a limited set of pathologies, including infection caused by RSV, a virus that is known to interfere with the interferon response (*33*, *34*), and bacterial sepsis that is characterized by a dysregulated host response to infection (*35*).

## DISCUSSION

Relatively little is known about the types of in vivo immune responses elicited by mRNA vaccines in humans. To address this, we used bulk blood transcriptomics to map the immune changes taking place in vivo after the administration of the first and second doses of COVID-19 vaccines in adult volunteers. We did so at a high temporal resolution, collecting small amounts of blood before and for nine consecutive days after the administration of the first and second doses of COVID-19 mRNA vaccines. The use of blood transcriptomics eliminated the need to choose a panel of analytes to measure vaccine responses, which is one source of bias. The daily collection and profiling schemes adopted eliminated the need to choose specific time points for measuring the response, thus eliminating a second source of bias.

**Fig. 9.**
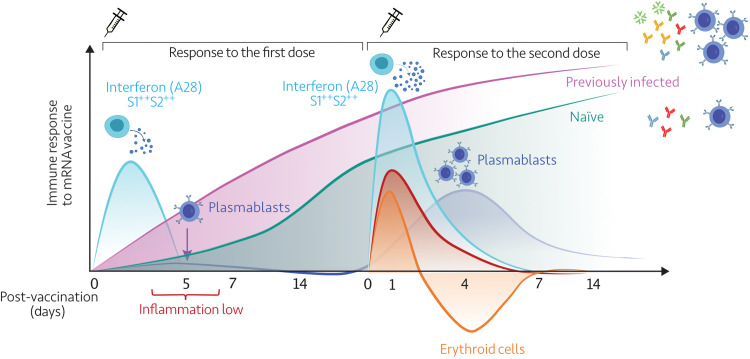
Summary. This diagrammatic representation summarizes the temporal trajectories of blood transcriptional signatures elicited in response to the first and second doses of mRNA vaccines.

Profiling blood transcript abundance after the first and second doses of COVID-19 mRNA vaccines at a high temporal resolution revealed a well-orchestrated sequence of immune events (Fig. 9). The immune signatures elicited following the administration of the two doses of mRNA vaccines differed drastically. Relatively modest changes were observed after the first dose that manifested primarily as the induction of interferon response signatures that were detectable over the first 3 days following the injection of the first dose. This was followed by a more subtle response that could be attributed to the priming of the adaptive response between days 4 and 6. A decrease in the abundance of transcripts for modules associated with inflammation was observed over these 3 days, which was accompanied by an increase in transcripts associated with plasma cells and T cells on day 5. No further changes were detected beyond day 6. After the second dose, the plasmablast response was more robust and peaked on day 4 but was not accompanied by a T cell response peak, as was the case after the first dose. Notably, in studies assessing blood transcriptional responses to vaccines, the peak plasmablast response is typically observed on day 7, as is the case, for instance, with influenza and pneumococcal vaccines (*6*, *26*, *27*). As a result, sampling schedules in common use that are designed to capture changes on days 1 and 7 and, sometimes, day 3 would miss the peak of the adaptive response to COVID-19 mRNA vaccines observed in our study.

In addition to eliminating potential blind spots, high-frequency sampling and profiling also permit the precise resolution of the complex kinetics of a response; for instance, the erythroid cell signature peaks sharply after the second dose and recedes well below baseline over several days before recovering. The trajectory of this signature may be of significance in the context of vaccination, as we recently described its association with immunosuppressive states, such as late-stage cancer and maintenance therapy in liver transplant recipients (*28*). In the same work, we found this signature to be strongly associated with the development of a more severe disease in subjects with acute respiratory syncytial virus infection. Notably, erythroid precursors have also been recently associated with more severe clinical outcomes in patients with COVID-19 (*36*). Populations of circulating erythroid cells have been found to have immunosuppressive properties (*37*). These properties may be exerted, for instance, through the expression by these cells of arginase or transforming growth factor–β, which have been found to suppress interferon-γ production in effector T cells and promote the development or regulatory T cells, respectively (*38*, *39*). Conversely, a recent report has shown that a defect in erythrocyte mitochondrial removal observed in patients with SLE was a driver for interferon production and correlated with disease activity (*40*). The downward trajectory followed by the A37/erythroid cell modules after the day 1 peak after the second dose was peculiar. It was not observed with the A35/inflammation module, for instance, which also peaked at day 1. It may be attributed to changes in relative cellular composition in the blood, reflecting either a mobilization of erythroid cells outside of the circulation or, conversely, a transient expansion of other populations of circulating leukocytes, resulting in a dilution of the erythroid cell population. Together, these observations warrant follow-up investigations into the potential role of circulating erythroid cells in the context of vaccination.

Arunachalam *et al.* (*5*) previously described the elicitation of qualitatively distinct innate signatures on day 1 following the administration of the first and second doses of COVID-19 mRNA vaccines, with the former inducing an interferon response and the latter a mixed response that also presented an inflammatory component. Our findings are consistent with these earlier observations and, using a high-frequency sampling and profiling protocol, permitted us to further dissect those responses. Most notably, while interferon responses appear a priori as the common denominator between the post–first and post–second dose responses, the temporal pattern of response that we observed indicates that these are qualitatively and quantitatively distinct. This was best evidenced by the differences in the timing of the response peak, which corresponded to day 2 after the first and day 1 after the second doses. The kinetics of the response after the second dose is therefore most consistent with what is observed following injection of a single dose of influenza vaccine (*6*). A further investigation of the patterns of response among the six modular components of the interferon responses (module aggregate A28) identified two distinct sets of modules. These two sets of three modules each, A28/S1 and A28/S2, displayed distinct kinetics and amplitude of response after the first and second doses. In an earlier report, we have shown that module repertoire analyses permitted the delineation of distinct interferon signatures and that those signatures could stratify patients with SLE (*30*), an observation that has since been confirmed in independent studies (*41*–*43*). We report here that distinct interferon signatures are elicited following vaccination. The delineation of these signatures was greatly aided by the adoption of a high–temporal resolution profiling approach. It permitted us to identify subtle but significant differences in amplitude and timing between the two signatures following both the first and second doses of mRNA vaccine. The response to the second dose appeared to be more potent and uniform across A28/S1 and A28/S2 modules and was accompanied by an increase in abundance of modules associated with inflammation and erythroid responses, among other signatures. Together, the differences observed after the first and second doses could be attributed to quantitative or qualitative differences, e.g., elicitation by the second dose of vaccine of the release of higher amounts of endogenous interferons or of the release of interferon of a different type (with the post–first dose signature possibly being elicited by type I interferons, as patterns of induction after interferon-β treatment would suggest, and the post–second dose signature possibly being elicited by type II interferon). The latter thesis is indirectly supported by recent findings in mice that received the Pfizer BioNTech BNT162b2 vaccine and were found to preferentially produce interferon-α in vivo after the first dose of vaccine and interferon-γ after the second dose (*44*). It should be, however, noted that while the pattern of induction of A28/S1 and S2 modules might be indicative of a given type of interferon response, our observations would not support the notion of a given set to be exclusively associated with a given type of response (i.e., the analyses of public datasets in fig. S7 show that A28/S1 modules may be more responsive to type I interferons, while the A28/S2 also appears to respond but only to a lesser degree).

Here, we also sought to determine whether “post–first dose–like” patterns (i.e., dominated by A28/S1, IRTP I) or “post–second dose–like” patterns (i.e., with potent induction of both components: A28/S1^++^ and A28/S2^++^, IRTP II) could be identified among patients with COVID-19. Since those were associated with the subsequent development of humoral immunity in the context of vaccination, it may be surmised that it would also be the case during the course of SARS-CoV-2 infection. This question was made particularly relevant in the context of COVID-19 disease, since it has been reported that failure to induce interferon responses is associated with worse disease outcomes (*8*, *45*–*47*). In the PREDICT-19 cohort, composed of patients with predominantly mild or moderate pathology, both phenotypes were observed, along with a third distinct phenotype that was not observed after vaccination. This latter phenotype is dominated instead by A28/S2, with A28/S1 abundance being low or even decreased (IRTP IIII). Notably, in a cohort of patients with severe disease, both A28/S1^++^ A28/S2^++^ (post–second dose–like/IRTP II) and A28/S2>S1 (IRTP III) phenotypes were also observed, with the latter being associated with extended lengths of stay in the ICU. However, IRTP III did not appear to be preferentially associated with death in this setting, which may be due to the supportive care provided to the patients. Overall, our observations are consistent with earlier work that has linked SARS-CoV-2 infection to impaired interferon responses and support the notion that failure to mount robust interferon responses is associated with a less favorable course of the disease (*8*, *48*). However, our findings also show that the response elicited by the infection in these patients may not be altogether defective (i.e., with only one component, A28/S1, being primarily affected). One possibility is that this peculiar response pattern may be associated with the presence of endogenously produced autoantibodies neutralizing interferon, as has been previously described (*47*, *49*), or, in a smaller proportion of individuals, of inborn errors of immunity (*46*, *50*). The high incidence of the IRTP IIII phenotype observed in patients with bacterial sepsis (about one in three), however, suggests that other mechanisms may be at play (e.g., suboptimal, delayed, or exhausted responses).

Other points remain to be elucidated. This includes the timing of the adaptive response to mRNA vaccines, which appears to rise and peak several days earlier than what is normally observed in responses to other vaccines (±7-day peak). The priming mechanism underpinning the robust polyfunctional response observed on day 1 after the second dose would deserve further investigation as well, with our findings suggesting that the first vaccine dose could contribute to the training of the innate immune response. Notably, the greater amplitude of responses observed after the second dose and the presence of an inflammatory component are also consistent with previous reports of the increase in the incidence of side effects/discomfort following the administration of the second dose of COVID-19 mRNA vaccine (*51*, *52*).

Last, while this study contributes to a better understanding of the drivers of mRNA vaccine immunogenicity, it can also serve as a resource to help inform the design of future studies investigating vaccine responses. A decrease in sequencing costs provides an opportunity to use transcriptome profiling approaches in novel ways. One of them is the implementation of high–temporal resolution profiling protocols. An advantage of the delineation of transcriptome responses at high temporal resolution is that it is doubly unbiased, i.e., there is no need to select transcripts for inclusion in a panel because RNA-seq measures all transcript species present in a sample. Similarly, there is no need to select specific time points for assessing the vaccine response, as all time points are profiled within a given time frame. Thus the approach permits the removal of potential blind spots and the detection of changes that may otherwise be missed by more sparse sampling protocols. In addition to eliminating potential blind spots, high-frequency profiling data helped resolve the vaccine response more precisely. This was the case in our study of the interferon response, with the delineation of two distinct components having been much more difficult if not for the precise resolution of peaks of response over the first 3 days after the first and second doses of vaccines. Some of the practical elements that may contribute to making the routine implementation of the high–temporal resolution transcriptomics approach viable include, as mentioned earlier, a substantial decrease in the cost of RNA-seq, especially 3′-biased methodologies. Along the same lines, recent publications showed through downsampling analysis that sequencing at read depths that are much shallower than is typical is adequate for biomarker discovery projects, which could lead to further reductions in the cost of RNA-seq assays (*53*). In turn, the lower costs should permit a substantial increase in sample sizes or, as in this case, sampling frequency. Another consideration is the availability of solutions for the in-home self-collection of samples. This is the case for the collection of RNA-stabilized blood with our custom method, which could be further improved. Novel solutions are also being put forward that could permit the implementation of these methods at scale (*54*). Last, as we have shown, it is possible to implement the self-collection of samples for serology profiling within a vaccinology study.

There were several limitations to our study. While the sample size was adequate for an initial discovery phase, a larger study cohort would help better resolve interindividual variations. The dataset that we generated, however, has been made available for reuse, and it should be possible to integrate and consolidate this dataset with those generated in follow-up studies by us and others (*28*). Follow-on studies would need to be designed to formally address specific questions, for instance, comparing responses in individuals who had previously been exposed to SARS-CoV-2 with those in naïve individuals. It would also be interesting to compare responses elicited by the Pfizer/BioNTech and Moderna vaccines, which was not possible in our study because of the small numbers of individuals that received the Moderna vaccine. Although we hoped that it would be possible to obtain more balanced sample sizes for a more detailed comparison, the speed at which the vaccinations were rolled out among our target population of health care workers meant that we had very little control over the number of volunteers that received the different types of vaccines or their status as naïve or previously exposed individuals. It would also have been particularly interesting to enroll patients from different age categories, especially the elderly population, but this again proved impossible.

In conclusion, the data presented here suggest that high–temporal resolution blood transcriptomics would provide a valuable means to precisely map and compare the types of responses elicited by the different types of COVID-19 vaccines. In addition, with several already approved and >20 currently in phase 3 trials (*55*), it could constitute a unique opportunity for the benchmarking of virtually all available vaccine platforms. Similarly, this approach could potentially be implemented to characterize and compare vaccine response profiles in populations that do not respond optimally to vaccines [e.g., in the elderly (*56*, *57*), immunosuppressed (*58*–*60*), and during pregnancy (*61*, *62*)]. This study also contributed to a better understanding of the drivers of mRNA vaccine immunogenicity and identified interferon signatures as early indicators of the potency of the humoral immune response elicited in individual subjects. It also led to the definition of functional interferon response phenotypes among patients with COVID-19 that were associated with different disease trajectories. In particular, mechanisms underlying the development of dysfunctional interferon responses remain to be elucidated, which may yield important insights into the pathogenesis of severe COVID-19 disease.

## MATERIALS AND METHODS

### Subject recruitment

#### COVAX cohort

We enrolled adult subjects eligible to receive a COVID-19 vaccine who were willing to adhere to the sampling schedule. The protocol was approved by Sidra Hospital Institutional Review Board (IRB) (IRB number 1670047-6), and all participants gave written informed consent. Inclusion criteria matched the clinical eligibility for receiving the vaccine, and the only exclusion criterion was to have received a first dose of any COVID-19 vaccine. Twenty-three subjects were enrolled, and the median age was 38 years (range, 29 to 57 years); 20 of the subjects received the Pfizer vaccine, and 3 received the Moderna vaccine. The demographics, health status at accrual, and vaccination side effects are shown in Table 1. Intervals between the first and second vaccine doses were typically 21 days for Pfizer and 29 days for Moderna.

#### IMPROVISE cohort

Adult subjects with severe COVID-19 were enrolled in this cohort under the Hamad Medical Corporation IRB approval (MRC-05-007). Blood samples were collected at multiple time points during the patients’ ICU stay (time point 1 was taken at ICU admission; time points 1 to 4 were 7 days apart). Subjects with burn and trauma, with immunological diseases, receiving immunosuppressive treatment, with other immune-related conditions, or with a previous COVID-19 infection were excluded. For this analysis, 40 patients with severe COVID-19 were included, with a median age of 52 years (range, 30 to 92 years). The clinical parameters of those patients included gender, ICU and hospital stay, mechanical ventilation duration, extracorporeal membrane oxygenation initiation, comorbidities, outcomes (death/recovery), nosocomial infection onset, and plasma therapy. Samples were also collected from control subjects who were adults and did not (i) present with an infectious syndrome during the past 90 days; (ii) experience extreme physical stress within the past week; (iii) receive, during the past 90 days, a treatment based on antivirals, antibiotics, antiparasitics, and antifungals; (iv) receive, within the past 15 days, a treatment based on nonsteroidal anti-inflammatory drugs; (v) receive, during the past 24 months, a treatment based on immunosuppressive therapy, corticosteroids, therapeutic antibodies, and chemotherapy; and (vi) have a history of innate or acquired immune deficiency, hematological disease, solid tumor, severe chronic disease, surgery or hospitalization within the past 2 years, pregnancy within the past year, participation to a phase 1 clinical assay during the past year, and participation to a phase 1 clinical assay during the past year; no samples were collected from pregnant or breastfeeding women and those with restricted liberty or under legal protection.

#### PREDICT-19 cohort

The “PREDICT-19” Consortium is an international consortium formed by a group of researchers who share common interests in identifying, developing, and validating clinical and/or bioinformatics tools to improve patient triage in a pandemic such as COVID-19 (*29*). The PREDICT-19 Italian cohort comprises adult subjects with mild, moderate, or severe COVID-19 diagnosed by real-time polymerase chain reaction on nasopharyngeal swab who were consented and enrolled at Ente Ospedaliero (E.O.) Ospedali Galliera and Istituto di Ricovero e Cura a Carattere Scientifico (IRCCS) Ospedale Policlinico San Martino, Genoa, Italy (Ethics Committee of the Liguria Region; N.CER Liguria 163/2020-ID 10475). Blood samples were collected during hospitalization. Subjects with burn and trauma, with immunological diseases, receiving immunosuppressive treatment for underlying disorders before COVID-19 diagnosis, with other immune-related conditions, or with a previous COVID-19 infection were excluded. Severity scores were determined at the time of sampling and on the basis of an eight-point system established by the World Health Organization (*63*). For this analysis, 10 healthy subjects and 99 patients with COVID-19 were included, with a median age of 61.76 years (range, 26 to 86 years).

### Sampling protocol

#### COVAX cohort

For transcriptomics applications for the COVAX study, after puncturing the skin with a fingerstick, 50 μl of blood was collected in a capillary/microfuge tube assembly supplied by KABE Labortechnik (Numbrecht, Germany) containing 100 μl of tempus RNA-stabilizing solution aliquoted from a regular-sized tempus tube (designed for the collection of 3 ml of blood and containing 6 ml of solution; Thermo Fisher Scientific, Waltham, MA, USA). This method is described in detail in an earlier report (*7*), and the collection procedure is illustrated in an uploaded video at www.youtube.com/watch?v=xnrXidwg83I (*64*). Blood was collected before the vaccine was administered (day 0), on the same day, and daily thereafter over the next 10 days. This protocol was followed for both the first and second vaccine doses. For serology applications, 20 μl of blood was collected using a Mitra blood collection device (Neoteryx, Torrance, CA, USA) before the vaccine was administered and on days 7 and 14 after vaccination with the first and second doses.

#### IMPROVISE cohort

For the IMPROVISE study, samples were collected using PaxGene Blood RNA tubes (BD Biosciences, Franklin Lakes, NJ, USA) at all time points and were frozen at −20°C until further processing.

#### PREDICT-19 cohort

For the Italian cohort of the PREDICT-19 study, blood samples were collected during hospitalization by venipuncture in tubes containing an RNA-stabilizing solution (Tempus Blood RNA Tube, Thermo Fisher Scientific, Waltham, MA, USA; catalog number 4342792) and frozen at −20°C until further processing.

### Multiplex serological assay

The presence of antibodies against selected human coronavirus proteins in the serum was measured with a home-built bead array based on carboxymethylated bead sets with six distinct intensities of an ultraviolet-excitable dye. Each bead set was individually coupled to the three SARS-CoV-2 proteins envelope, nucleoprotein, and Spike protein in its trimeric form or its fragments and the S1 fragment of SARS-CoV S protein. Therefore, the complete array consisted of six antigens, including five SARS-CoV-2 antigens (Full Spike Trimer, Receptor Binding Domain, Spike S1, Nucleoprotein, and Envelope), as well as the closely related SARS-CoV-S1 protein. The binding of human antibodies to each viral antigen (bead set) is revealed with fluorescently labeled isotype-specific mouse monoclonal or polyclonal antibodies. We measured total IgM, total IgG, and total IgA as well as their individual isotypes IgG1, IgG2, IgG3, IgA1, and IgA2, reporting a total of 48 parameters per sample. The assays were performed on filter plates and acquired on a BD Symphony A5 using a high-throughput sampler. An average of 300 beads per region was acquired, and the median fluorescence intensity (MFI) for each isotype binding was used for characterizing the antibody response. An antibody response index was calculated as the ratio of the MFI of pooled negative blood controls collected before June 2018 (Sidra IRB 1609004823) to the MFI obtained for vaccinated donor samples.

### RNA extraction and quality control

RNA was extracted using the Tempus Spin RNA Isolation Kit (Thermo Fisher Scientific), which was adapted for the handling of small blood volumes. The methodology has been described previously in detail (*65*). Contaminating DNA was removed using the TurboDNAse kit (Thermo Fisher Scientific), and RNA was quantitated on a Qubit instrument (Thermo Fisher Scientific) and quality controlled using an Agilent 2100 Bioanalyzer (Agilent, Santa Clara, CA, USA).

### RNA sequencing

#### COVAX and IMPROVISE cohorts

mRNA sequencing was performed using the QuantSeq 3′ mRNA-Seq Library Prep Kit FWD for Illumina (75 single end) with a read depth of 8M and an average read alignment of 79.60%. Single samples were sequenced across four lanes, and the resulting FASTQ files were merged by sample. Quality trimming is performed to remove adapter sequences and polyadenylate tails. Then, trimmed reads were aligned to human genome GRCh38/hg38 (Genome Reference Consortium Human Build 38, International Nucleotide Sequence Database Collaboration (INSDC) Assembly GCA_000001405.28, December 2013) using STAR 2.6.1d, and featureCounts v2.0.0 was used to generate the raw counts. Raw expression data were normalized to size factor effects using the R package DESeq2. All downstream analyses were performed using R version 4.1 unless otherwise specified. Global transcriptional differences between samples were assessed by principal components analysis using the “prcomp” function. Transcriptome profiling data were deposited, along with detailed sample information, into a public repository, the National Center for Biotechnology Information (NCBI) GEO, with accession ID GSE190001 and BioProject ID PRJNA785113.

#### PREDICT-19 cohort

Total RNA was isolated from whole-blood lysate using the Tempus Spin Isolation kit (Applied Biosystems) according to the manufacturer’s instructions. Globin mRNA was depleted from a portion of each total RNA sample using the GLOBINclear-Human kit (Thermo Fisher Scientific). Following the removal of globin transcripts, transcriptome profiles were generated via mRNA sequencing using Illumina HiSeq 4000 Technology (75 paired end) with a read depth of 60M. Single samples were sequenced across four lanes, and the resulting FASTQ files were merged by sample. All FASTQs passed quality control and were aligned to reference genome GRCh38 using STAR (2.6.1d). BAM files were converted to a raw count’s expression matrix using HTSeq (https://zenodo.org/record/6985383#.YvZa7uxBz0o). Raw count data were normalized using DESeq2. The ensemble IDs targeting multiple genes were collapsed (average), and a final data matrix gene was generated for modular repertoire analysis.

### Statistical analysis

Analyses were conducted using predefined gene sets. Specifically, we used a fixed repertoire of 382 transcriptional modules that were thoroughly functionally annotated, as described in detail in a recent publication (*14*). Briefly, this repertoire of transcriptional modules (“BloodGen3”) was identified on the basis of coexpression, as measured in a collection of 16 blood transcriptome datasets encompassing 985 individual transcriptome profiles. Sets of coexpressed transcripts were derived from the analysis of a large weighted coclustering network. Downstream analysis results and visualizations were generated using a custom R package (*66*). The workflow consists of, first, annotating the expression matrix (DESeq2-normalized counts) with module repertoire information (mapping transcripts to BloodGen3 modules); second, determining differential expression, which, as detailed below, can be done at either the level of groups or individual samples; and third, calculating the “module response,” which is defined as the percentage of constitutive transcripts with a given abundance that was determined to be different between two study groups or for the same individual in comparison to a given baseline (in this study, prevaccination abundance levels). The values, therefore, ranged from 100% (all constitutive transcripts increased) to −100% (all constitutive transcripts decreased). Only the dominant trend (i.e., increase or decrease in abundance over control/baseline) was retained for visualization purposes on fingerprint grids or fingerprint heatmaps, with red indicating an increase and blue indicating a decrease in abundance. When performing group comparisons (e.g., cases versus controls for the disease datasets used as reference), the *P* value and FDR cutoffs were applied (DESeq2 FDR < 0.1). When performing longitudinal analyses, the module response is determined by using fixed fold change (FC) and expression difference cutoffs (|FC| > 1.5 and |DIFF| > 10). Significance was determined for each module using the differential gene set enrichment function of the dearseq R package (*15*). The BloodGen3Module package was also used for visualizing module response. In the case of the vaccine cohort: (i) using fingerprint grid plots representing module response for a group of subjects (previously exposed and naïve) at a given time point in comparison to the prevaccination baseline (before the first or before the second dose, as applicable), as is the case for Figs. 2A, 3A, 4, 5A, and 6A), or (ii) using fingerprint heatmaps representing module response for individual subjects compared to their prevaccination baseline, as is the case for Figs. 2B and 5B. In addition, line graphs in Figs. 2, 4, and 5 showing average individual response over time for a set of modules from a given aggregate were generated using the ggplot2 R package (*67*). Bar graphs showing the number of responsive modules in Figs. 2A, 3A, 4A, and 6A were generated using the same package. In these instances, a module is considered responsive when the dominant proportion of constitutive transcripts reaches a defined threshold, which was set to 15% as to limit permissiveness to noise. To calculate correlations between module response levels and serum antibody levels before vaccination and at specific time points after vaccination, we used instead single-sample gene set enrichment analysis that was implemented in the GSVA package (*68*), and enrichment scores of individual samples were used for the Spearman correlation analysis (Figs. 2, 5, and 6 and figs. S2, S3, and S9 to S13). This approach permitted us to assign a module response value to prevaccination samples, which was not possible using the BloodGen3Module package since it uses these samples as a baseline for calculating module responses after vaccination. Spearman correlation results are shown in Figs. 2, 5, and 6 and figs. S2, S3, S9 to S13. We additionally performed linear mixed-effect modeling of the module activity (single-sample gene set enrichment analysis score) according to antibody index levels at baseline (before the first or second vaccination) and day 7 or 14 following vaccination and infection history. We have two observations per patient (at baseline and any given day after vaccine for transcriptional profiling data and at baseline and day 7 or 14 after vaccination for the antibody index), and we account for repeated measurements through a patient random effect on the intercept. The slope *P* value indicated on the scatterplots shown in figs. S2 and S9 to S13 characterizes the significance of the association between module activity and the antibody index.

### Definition of IRTPs

Study cohorts were stratified on the basis of patterns of interferon response for two distinct interferon signatures, defined as A28/S1 (comprising modules M8.3, M10.1, and M15.127) and A28/S2 (comprising modules M13.17, M15.64, and M15.86). For this, phenotypes were defined on the basis of levels of response observed for these two traits, as follows: Percentage responses of the six interferon modules were scored on the basis of the degree of response (% response ≥ 50, score = 2; 0 < %response < 50, score = 1; % response ≤ −50, score = −2; −50 < % response < 0, score = −1). Then, the average scores of S1 (“M8.3,” “M10.1,” and “M15.127”) and S2 (“M13.17,” “M15.64,” and “M15.86”) and phenotypes were classified using cutoff at S1/S2^++^ (average score ≥ 1), S1/S2^+^ (1 < average score < 0.33), S1/S20 (0.33 < average score ≤ 0), and S1/S2^−^ (average score < 0). The phenotypes were grouped as follows:

1) “IRTP I” = IRTP I = A28/S1^++^A28/S2^+^, A28/S1^++^A28/S2^0^, and A28/S1^+^A28/S2^0^

2) “IRTP II” = A28/S1^++^A28/S2^++^”

3) “IRTP III” = A28/S1^−^A28/S2^++^, A28/S1^−^A28/S2^+^, A28/S1^0^A28/S2^++^, and A28/S1^0^A28/S2^+^

4) The “other” category encompassed the remaining phenotypes = A28/S1^+^A28/S2^0^, “A28/S1^0^A8/S2^+^,” “A8/S1^+^A28/S^2^,” “A28/S1^0^A28/S2^0^,” “A28/S1^0^A28/S2^−^,” “A28/S1^−^A28/S2^−^,” “A28/S1^+^A28/S2^++^,” “A28/S1^+^A28/S2^+^,” and “A28/S1^−^A28/S2^0^”

## Supplementary Materials

### This PDF file includes:

Figs. S1 to S15 Membership of the PREDICT-19 consortium

## Notes

View/request a protocol for this paper from *Bio-protocol*.
