# Supplementary material for: High–temporal resolution profiling reveals distinct immune trajectories following the first and second doses of COVID-19 mRNA vaccines
Source: Sci Adv. 2022 Nov 11;8(45):eabp9961. doi: 10.1126/sciadv.abp9961 (PMC9651857; doi:10.1126/sciadv.abp9961)
Supplement: Supplementary file 1 — Figs. S1 to S15 Membership of the PREDICT-19 consortium [file sciadv.abp9961_sm.pdf]

Science Advances
AAAS

# Supplementary Materials for

## High–temporal resolution profiling reveals distinct immune trajectories following the first and second doses of COVID-19 mRNA vaccines

Darawan Rinchai *et al.*

Corresponding author: Darawan Rinchai, drinchai@rockefeller.edu;
Damien Chaussabel, damien.chaussabel@jax.org

*Sci. Adv.* **8**, eabp9961 (2022)
DOI: 10.1126/sciadv.abp9961

**The PDF file includes:**

Figs. S1 to S15
Membership of the PREDICT-19 consortium
Legends for supplemental files S1 to S4

**Other Supplementary Material for this manuscript includes the following:**

Supplemental files S1 to S4

## Supplementary Figures:

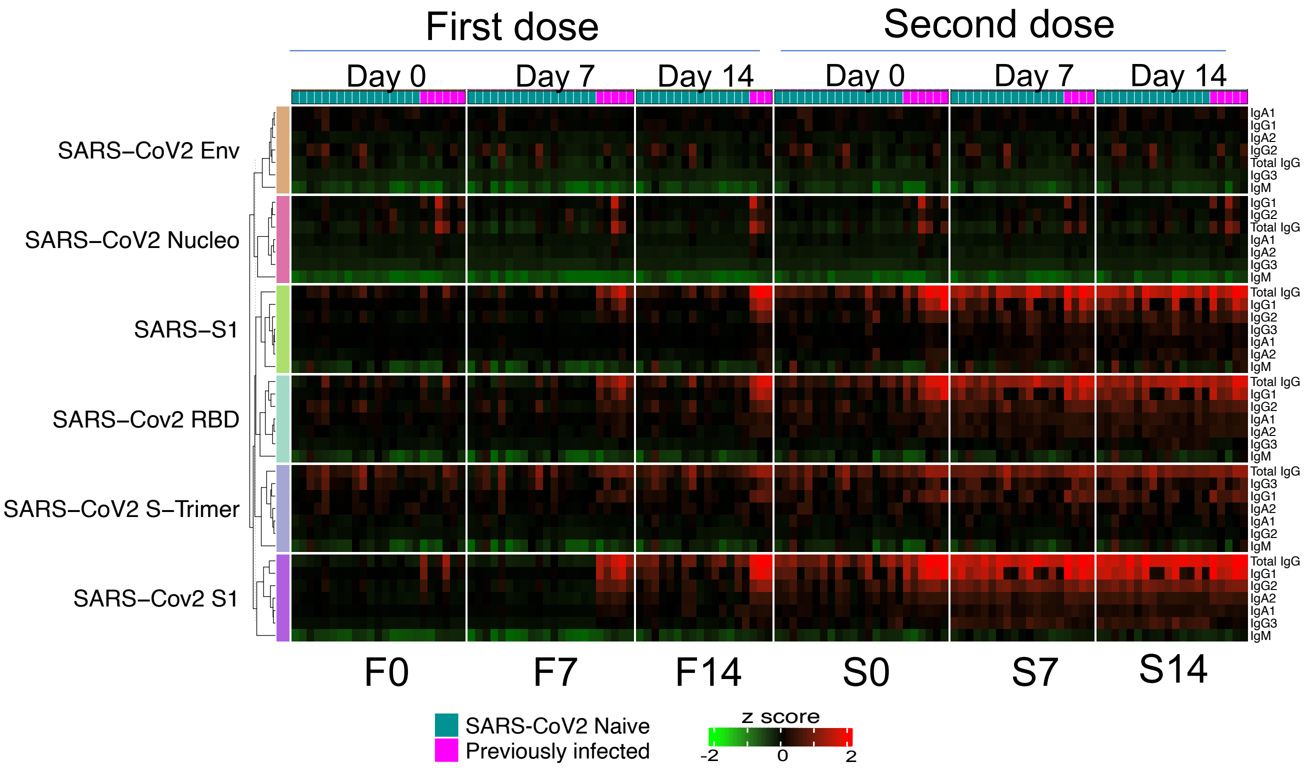


**Figure S1: Antibody response to COVID-19 mRNA vaccination (shown as an antibody index):** The heatmap represents the antibody index computed for individual subjects across specificities and antibody types. Red indicates a relative increase, and green indicates a relative decrease in abundance. Columns represent subjects arranged by timepoint with a colored track at the top indicating whether the subjects were naïve or had previously been infected with SARS-CoV-2. The rows represent antibody isotypes arranged by antigen specificity.

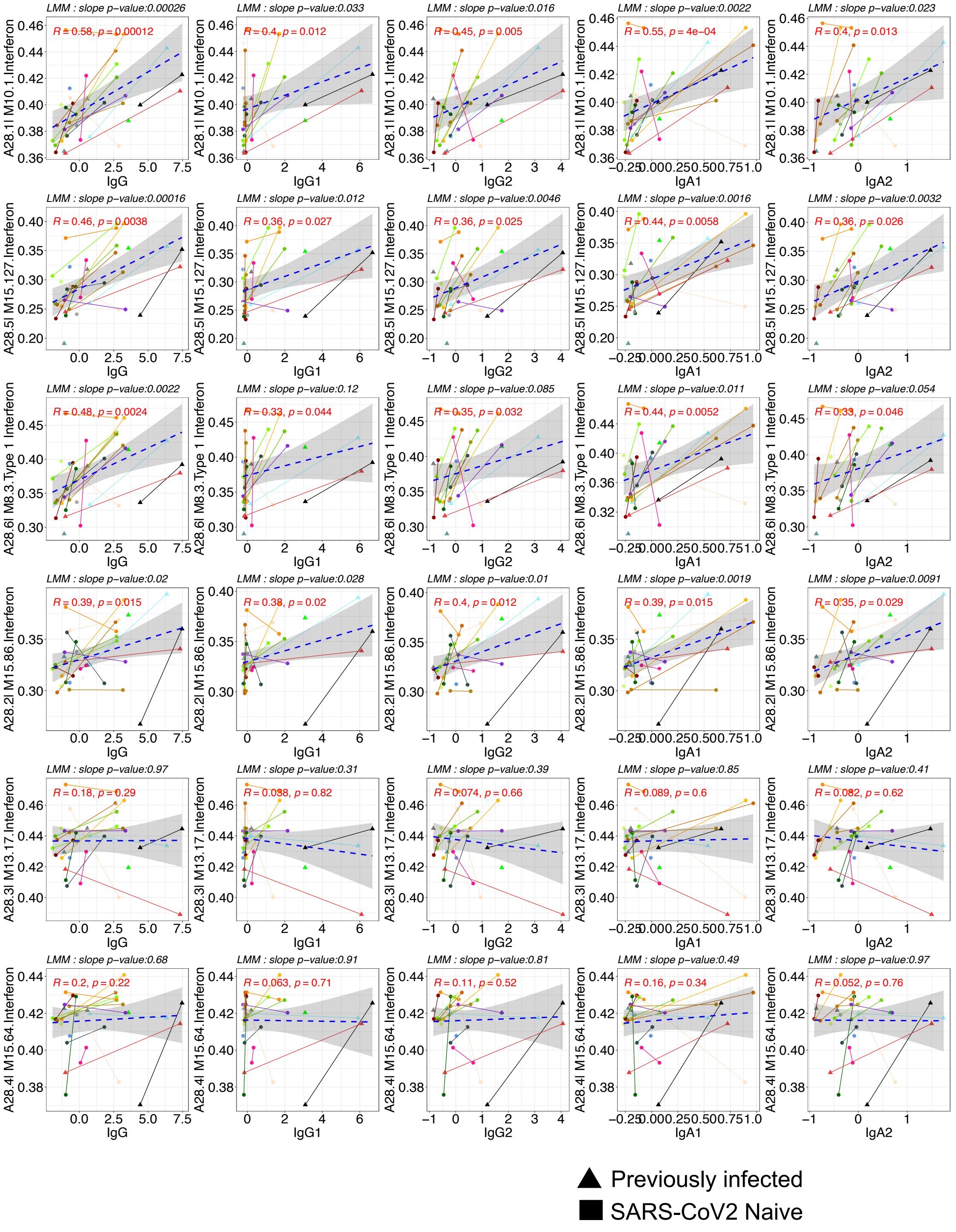


**Figure S2: Association of SARS-CoV2 S1 specific antibody responses measured on day 14 post first-dose with A28/interferon response measured on day 2 post-first dose:** The correlation plots indicate the degree of association between interferon module scores (Y-axis: computed as single sample gene enrichment score) and antibody index (X-Axis). Spearman’s correlation R value and p-value are shown on each of the plots, along with slope p-value (from linear mixed-effect modeling - see methods for details). One baseline pre-vaccine and one post-vaccine time

point are shown for each individual subject (color-coded by donor and connected by a line. In addition: squares = SARS-CoV2 Naïve, triangles = previously infected and recovered). Results are shown for 6 different interferon modules (rows) across multiple antibody isotypes (columns).

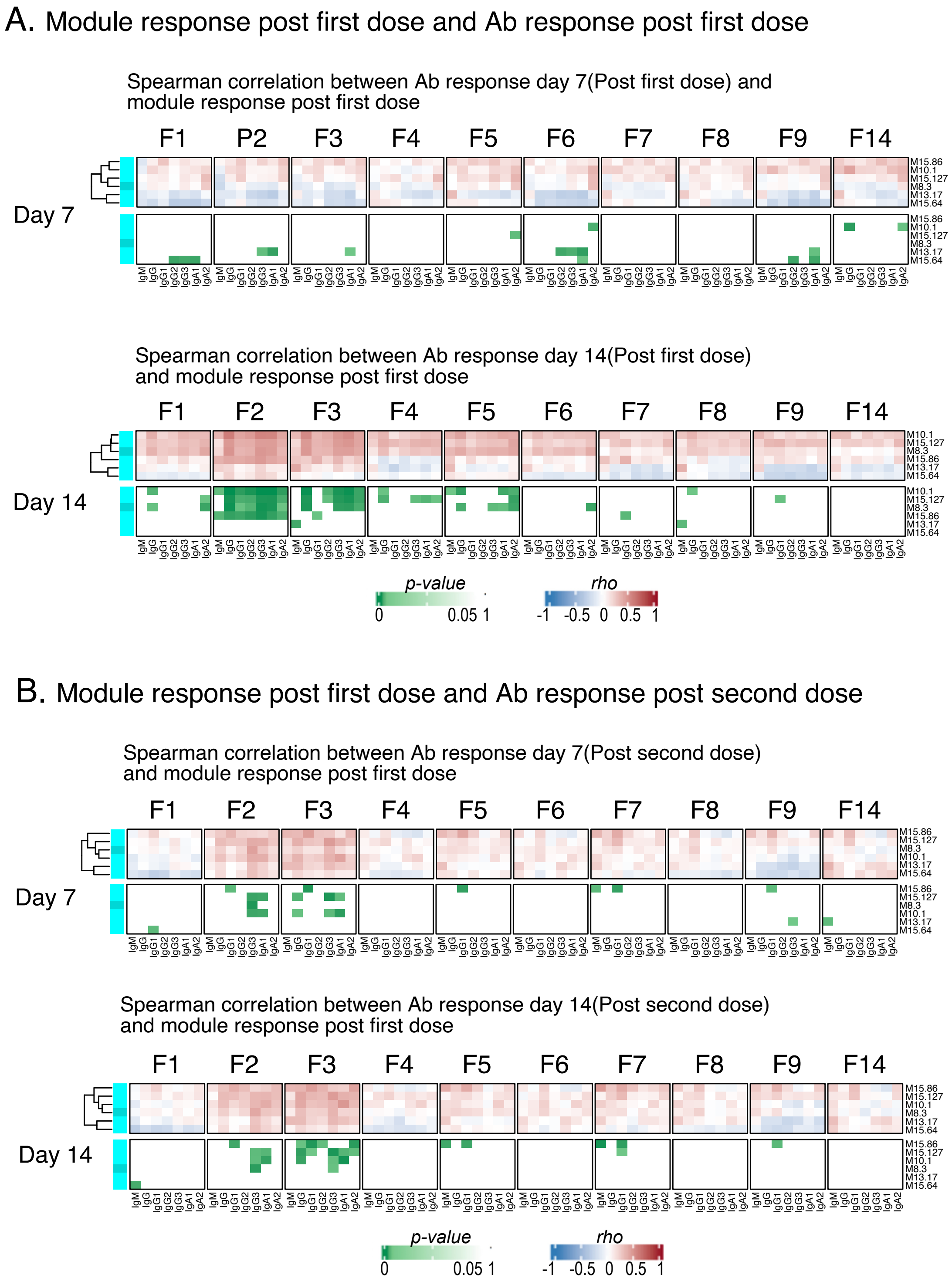


**Figure S3: Association of SARS-CoV2 S1 specific antibody responses measured post-first dose and post-second dose with A28/interferon response measured on 10 different days post-first dose.** The heatmaps represent Spearman's correlation between levels of module response measured at the pre-vaccination baseline (F0) and for each time points post-first dose (F1-9, F14)

and SARS-CoV2 S1-specific antibody levels measured at the pre-vaccination baseline (F0) and at 7 or 14 days post-first (F7, F14) or post-second dose (S7, S14). For each display item heatmaps at the top (blue-red color gradient) show the correlation coefficients with, as columns, days post-first vaccination (F1-F14) grouped by days across multiple antibody types, and, as rows, the six A28 interferon modules. The heatmap below (green color gradient) represents the significance of the correlations shown on the heatmap directly above, with the same ordering of rows and columns. (A) Shows correlation with antibody responses measured at day 7 and 14 post-first dose. (B) Shows correlation with antibody responses measured at day 7 and 14 post-second dose.

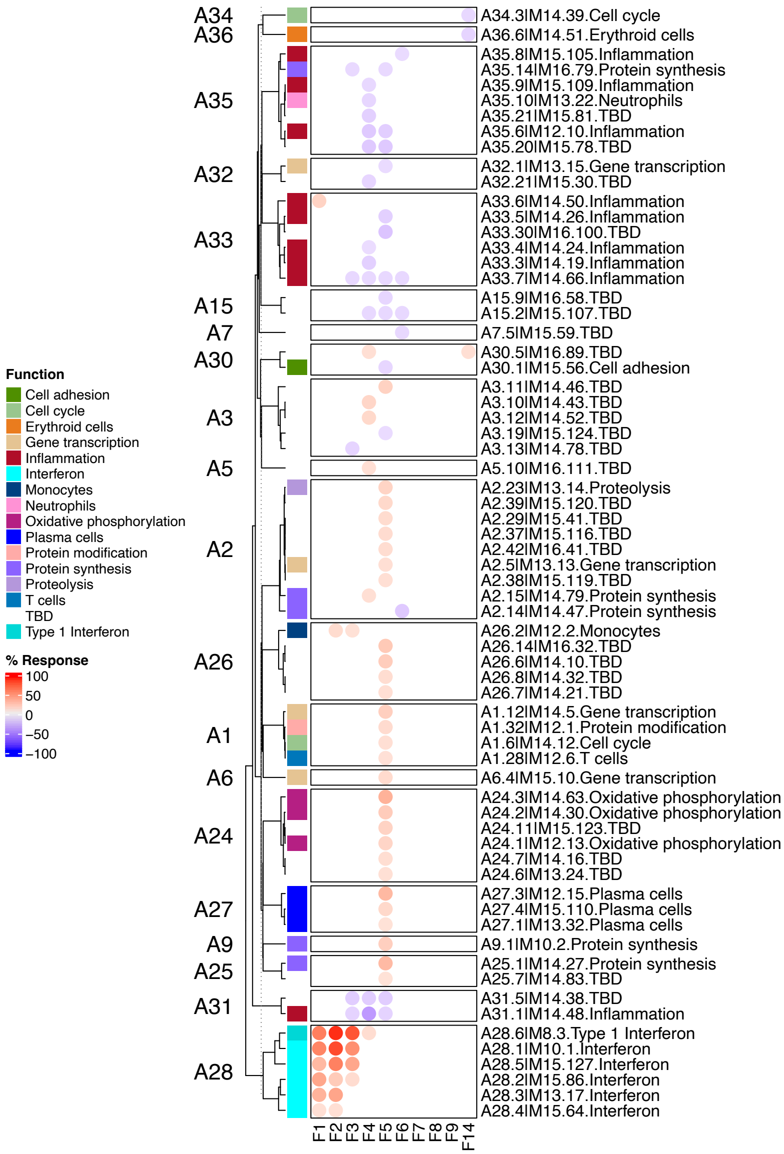


**Figure S4: Group-level modular blood transcriptional response post-first dose.** This fingerprint heatmap represents the module response post-first dose plotted at each time point post-vaccination. The modules are arranged as rows, and grouped by aggregates (A1, A2, etc..). Time points post vaccines are ordered according to days post-vaccination (F1 = day 1 post-first dose, F2 = day 2 post-first dose, etc…). The color track indicates module functional annotations. The module names include grid position (A34.3 = row A34, column 3), module identifier (M14.39 = 39th module formed as part of the 14th round of selection), and annotation. The colored spots of varying intensity represent the module response, with red spots indicating that transcripts constituting a given module are found to be predominantly increased in comparison to the pre-vaccine baseline, and blue spots indicating that transcripts are predominantly decreased (FDR<0.1).

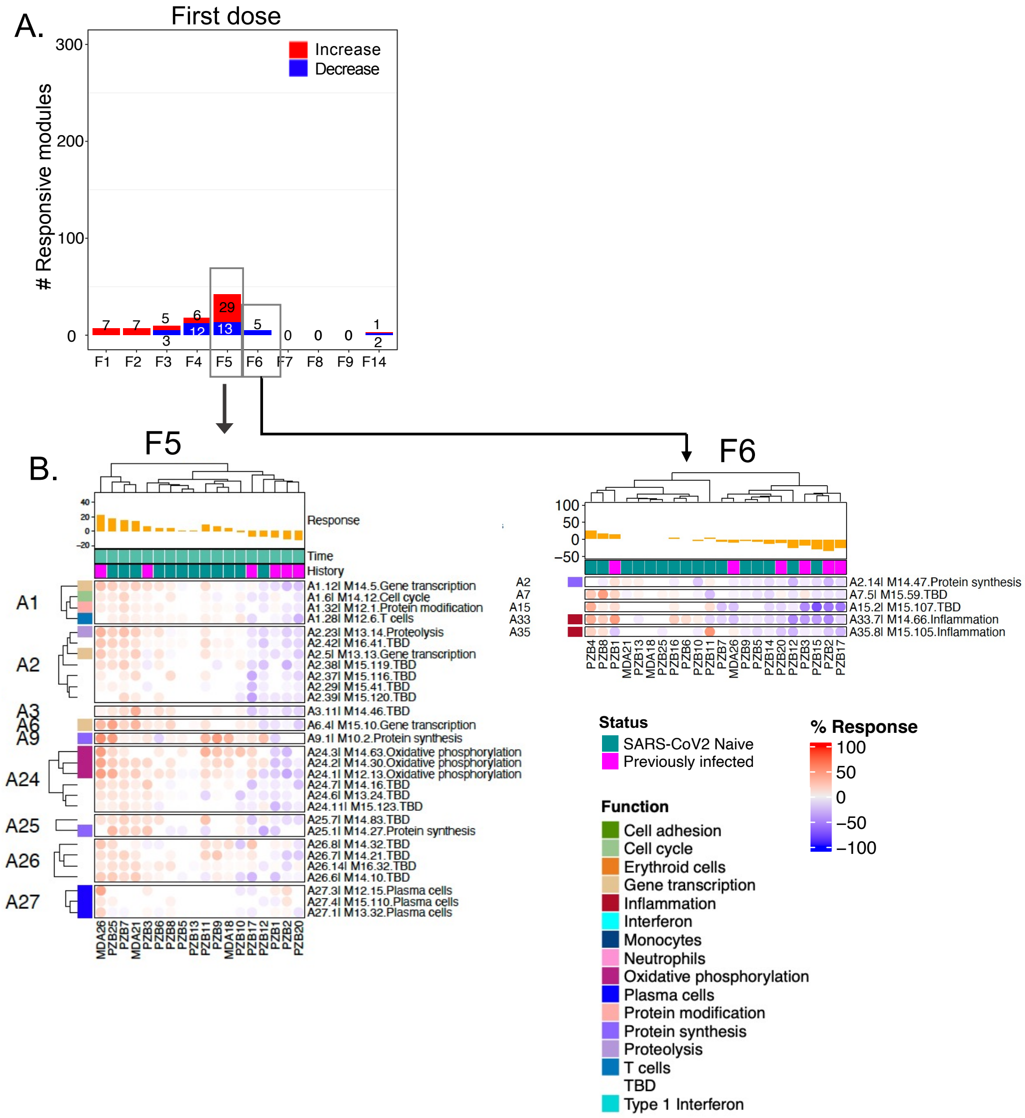


**Figure S5: Individual-level modular blood transcriptional response at days 5 and 6 post-first dose. (A)** The bar graph shows the cumulative module response at different days following the administration of the first dose of the vaccine (noted F1-F14). The Y-axis values and numbers on the bars indicate the number of modules meeting the 15% response threshold (out of a total of 382 modules constituting the BloodGen3 repertoire, with percentage response corresponding to the proportion of transcripts predominantly increased or decreased compared to baseline using DESeq2 FDR < 0.1 as the cutoff to determine significance). The number of modules for which abundance was predominantly increased is shown in red, and those for which abundance was predominantly decreased are shown in blue. **(B)** The fingerprint heatmaps represent the module response of individual subjects observed on days 5 and 6 following the administration of the first

dose of Covid-19 mRNA vaccine (F5 and F6, respectively). The modules are arranged as rows, and grouped by aggregates (A1, A2, etc...), subjects are arranged as columns and arranged by hierarchical clustering. The panel above (bar graph) shows for each individual the average response across all modules shown on the heatmap. The vertical color track indicates module functional annotations. The horizontal track indicates whether subjects are naïve or recovered from previous infection by SARS-CoV2. The colored spots of varying intensity represent the module response, with red spots indicating that transcripts constituting a given module are found to be predominantly increased in comparison to the pre-vaccine baseline (FDR<0.1), and blue spots indicating that transcripts are predominantly decreased.

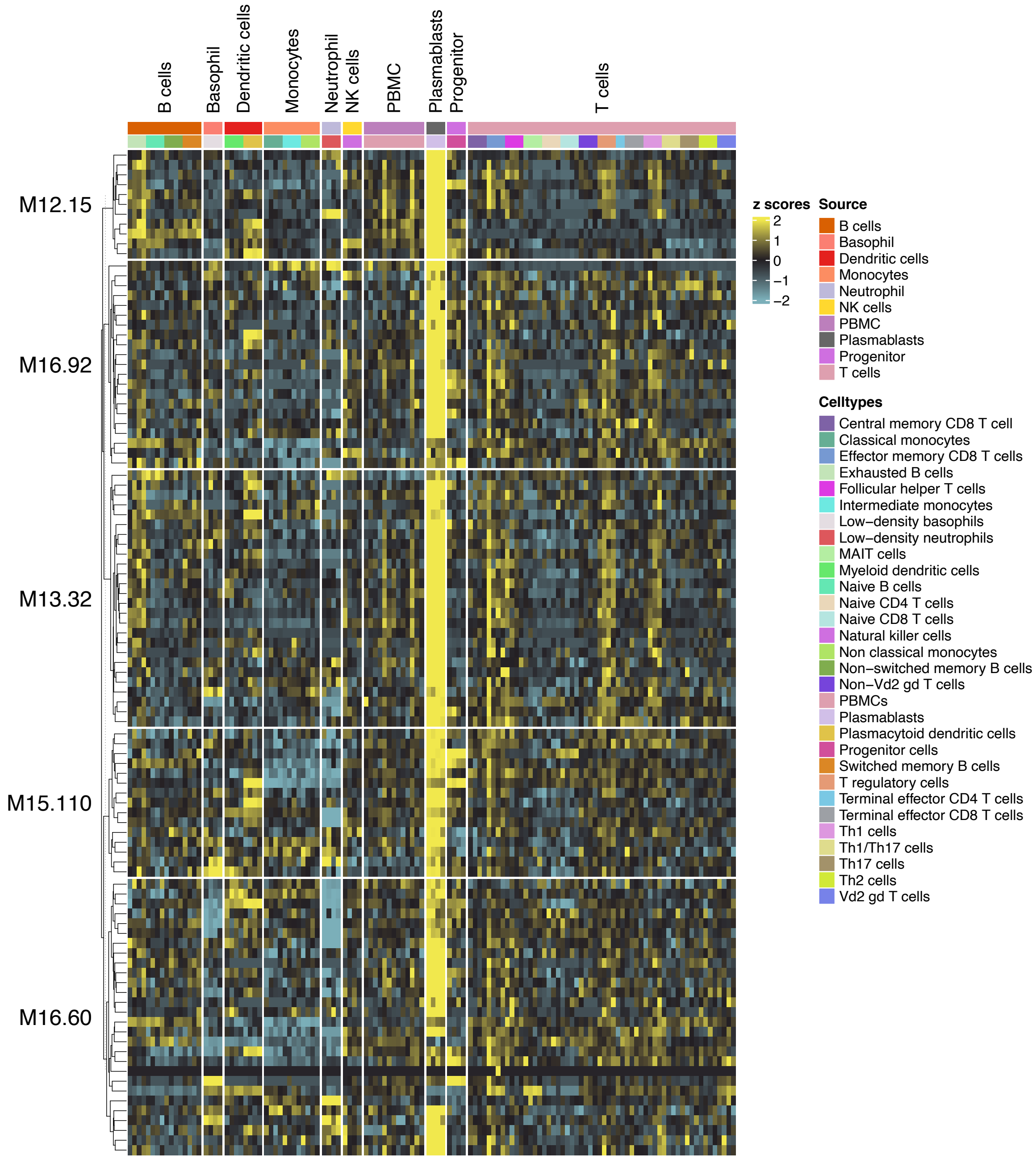


**Figure S6: Expression levels of A27 genes across cell populations isolated from human peripheral blood.** The abundance levels of transcripts comprised in the five modules constituting A27 (rows) across blood-cell populations (columns). The horizontal tracks at the top indicate cell types. A27 modules can be explored interactively here: https://prezi.com/view/GgIiA0K9kSFHbpVj2I85/. The dataset was contributed by Monaco et al *(20)* and is publicly available under GEO accession ID: GSE107011.

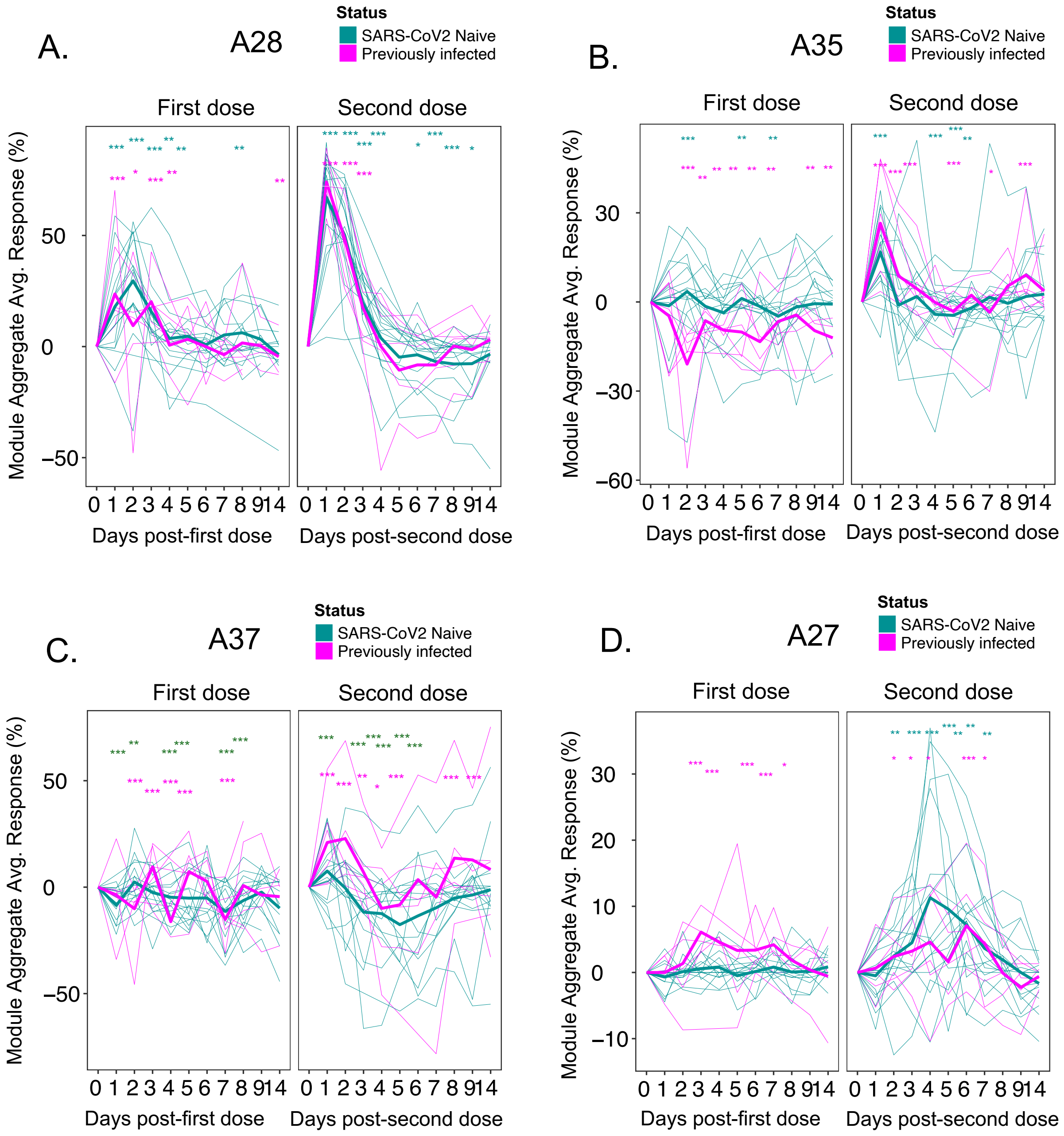


**Figure S7: Individual subjects module aggregate-level responses post-first and post-second vaccine dose.** Each line on those graphs represents for an individual subject the percentage response averaged for all the modules within a given aggregate, across all time points post-first or post-second vaccine dose. Responses are shown for aggregates A28 (Interferon response, panel A.), A35 (Inflammation, panel B.), A37 (Erythroid cells, panel C.) and A27 (Plasmablasts, panel D.). The teal and pink bolded lines represent averaged response for SARS-CoV2 naïve and previously infected individuals, respectively. For each module aggregate we also determined the significance of changes measured post-vaccination and at each time point (paired t-test

comparing module response at each time point relative to the pre-vaccination baseline separately for naïve and recovered subjects. For all tests: * p<0.01, ** p<0.001, *** p<0.0001.

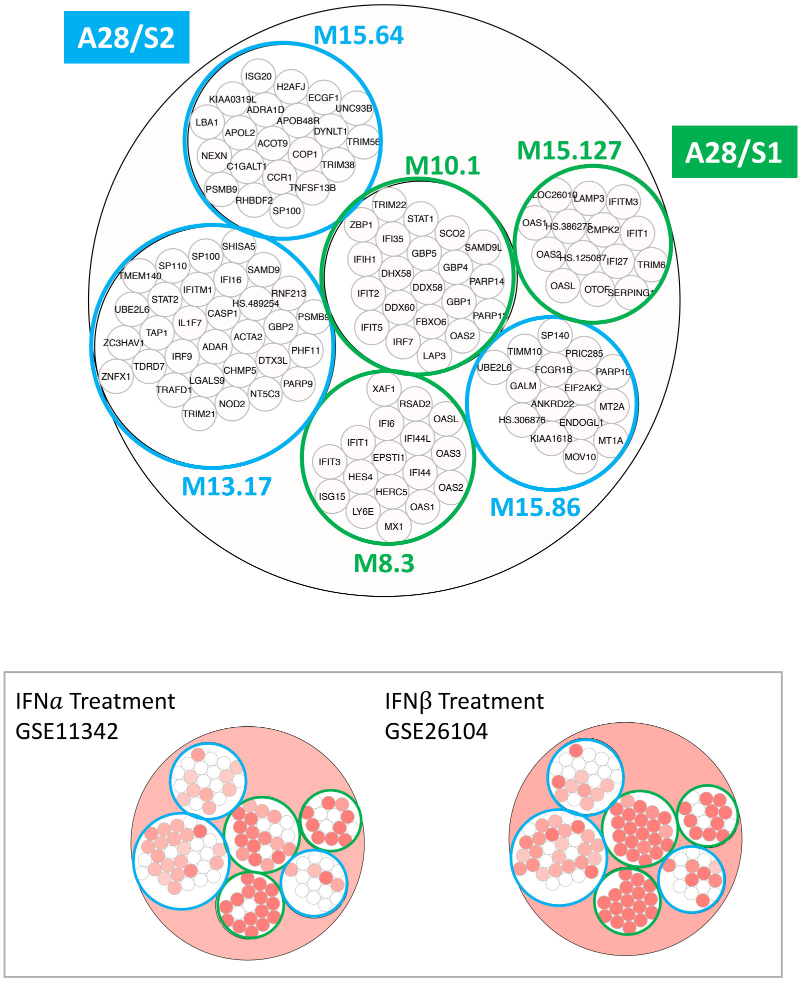


**Figure S8: In vivo changes in transcript abundance across the six A28 interferon-response modules post-treatment with type I interferon.** The circle packing plot at the top shows the gene composition of each of the six A28 modules, with A28/S1 modules highlighted in green and A28/S2 modules highlighted in blue. The smaller circles at the bottom show changes in abundance of A28 transcripts for two public datasets. One study (GSE26104) measured transcriptional response in subjects with multiple sclerosis treated with beta-interferon *(31).* The second study (GSE11342) measured blood transcriptional response in patients with Hepatitis C infection treated with alpha-interferon *(32).* A red circle indicates a significant increase in the abundance of a given transcript compared to the pre-treatment baseline (FDR < 0.1).

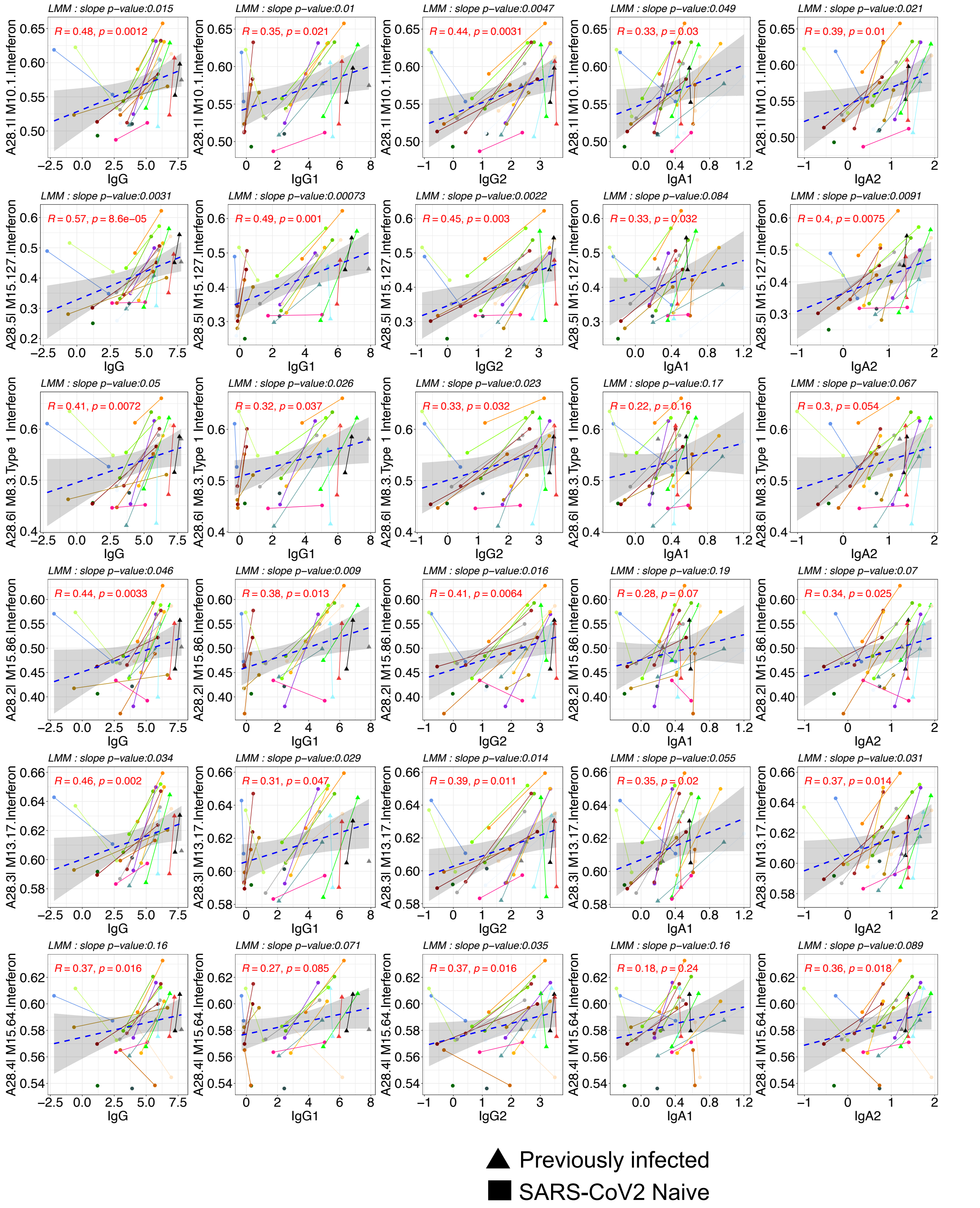


**Figure S9: Association of SARS-CoV2 S1 specific antibody responses measured on day 14 post second-dose with A28/interferon response measured on day 1 post-second dose:** The correlation plots indicate the degree of association between interferon module scores (Y-axis: computed as single sample gene enrichment score) and antibody index (X-Axis). Spearman's

correlation R value and p-value are shown on each of the plots, along with slope p-value (from linear mixed-effect modeling - see methods for details). One baseline pre-vaccine and one post-vaccine time point are shown for each individual subject (color-coded by donor and connected by a line. In addition: squares = SARS-CoV2 Naïve, triangles = previously infected and recovered). Results are shown for 6 different interferon modules (rows) across multiple antibody isotypes (columns).

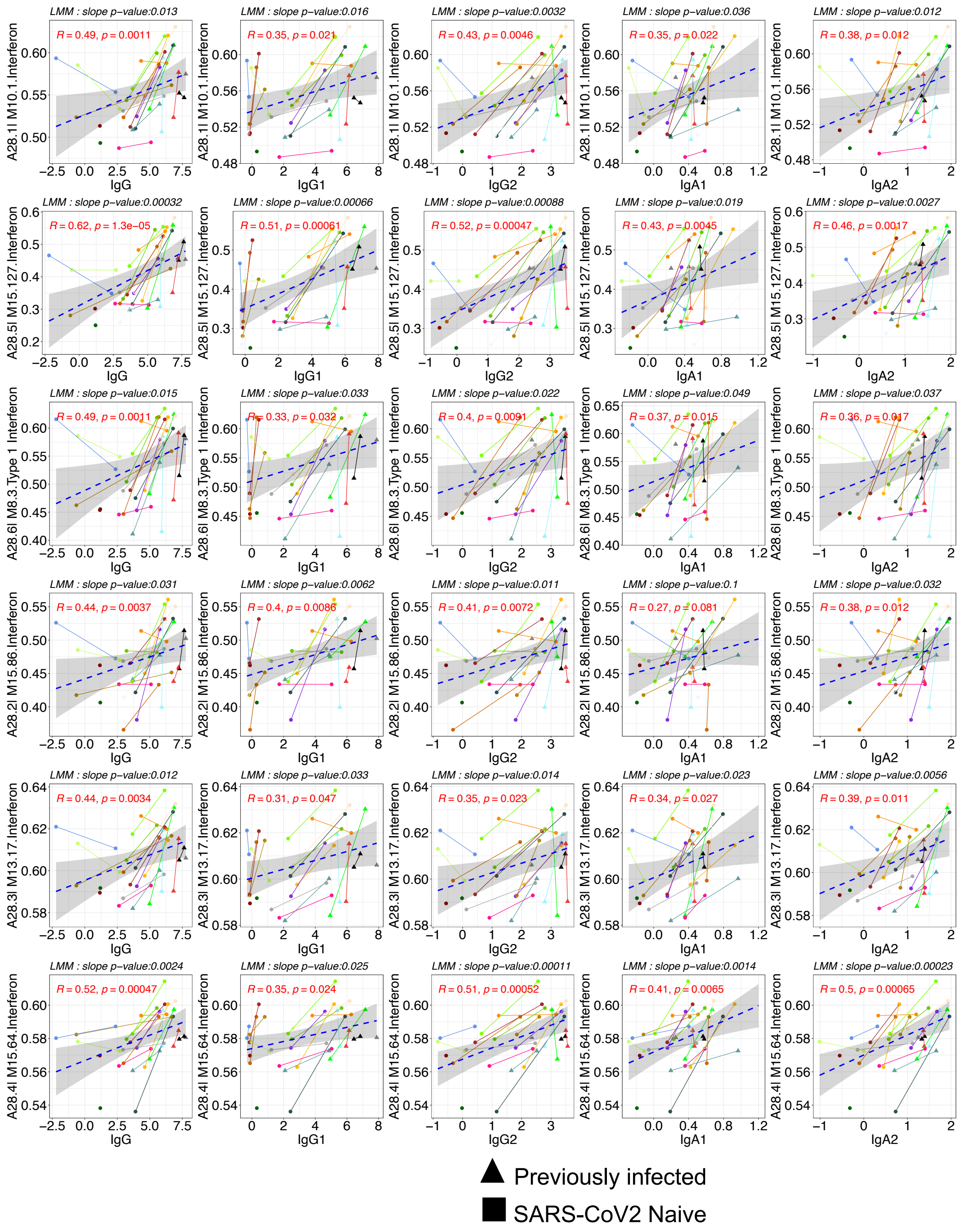


**Figure S10: Association of SARS-CoV2 S1 specific antibody responses measured on day 14 post second-dose with A28/interferon response measured on day 2 post-second dose:** The correlation plots indicate the degree of association between interferon module scores (Y-axis: computed as single sample gene enrichment score) and antibody index (X-Axis). Spearman's correlation R value and p-value are shown on each of the plots, along with slope p-value (from linear mixed-effect modeling - see methods for details). One baseline pre-vaccine and one post-

vaccine time point are shown for each individual subject (color-coded by donor and connected by a line. In addition: squares = SARS-CoV2 Naïve, triangles = previously infected and recovered). Results are shown for 6 different interferon modules (rows) across multiple antibody isotypes (columns).

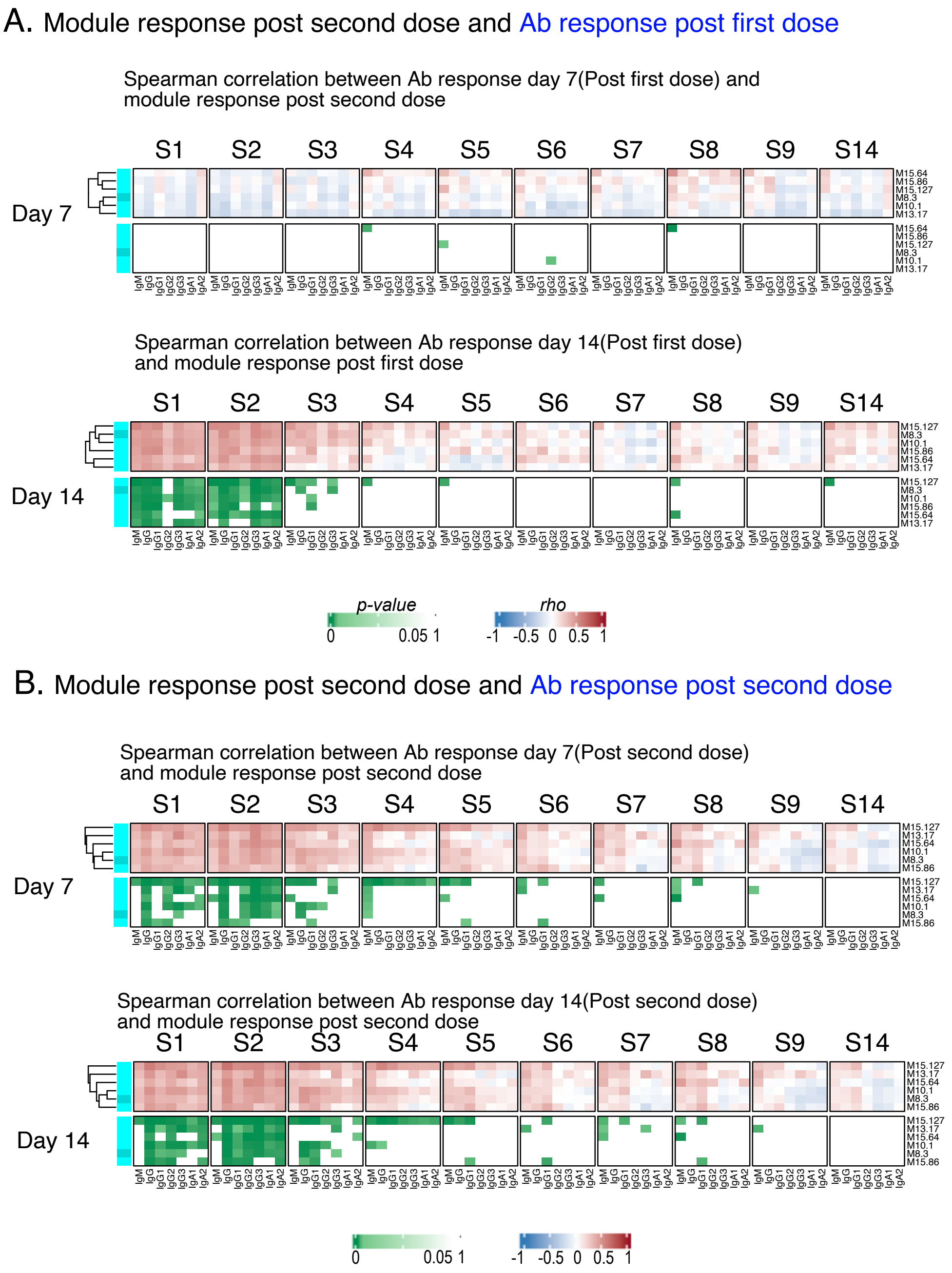


**Figure S11: Association of SARS-CoV2 S1 specific antibody responses measured post-first dose and post-second dose with A28/interferon response measured on 10 different days post-second dose.** The heatmaps represent Spearman's correlation between levels of module response measured at the pre-vaccination baseline (S0) and for each time point post-second dose (S1-9, S14) and SARS-CoV2 S1-specific antibody levels measured at the pre-vaccination baseline (S0) and at 7 or 14 days post-first (F7, F14) or post-second dose (S7, S14). For each display item heatmaps at the top (blue-red color gradient) show the correlation coefficients with, as columns,

days post-first vaccination (S1-S14) grouped by days across multiple antibody types, and, as rows, the six A28 interferon modules. The heatmap below (green color gradient) represents the significance of the correlations shown on the heatmap directly above, with the same ordering of rows and columns. (A) Shows correlation with antibody responses measured at day 7 and 14 post-first dose. (B) Shows correlation with antibody responses measured at day 7 and 14 post-second dose.

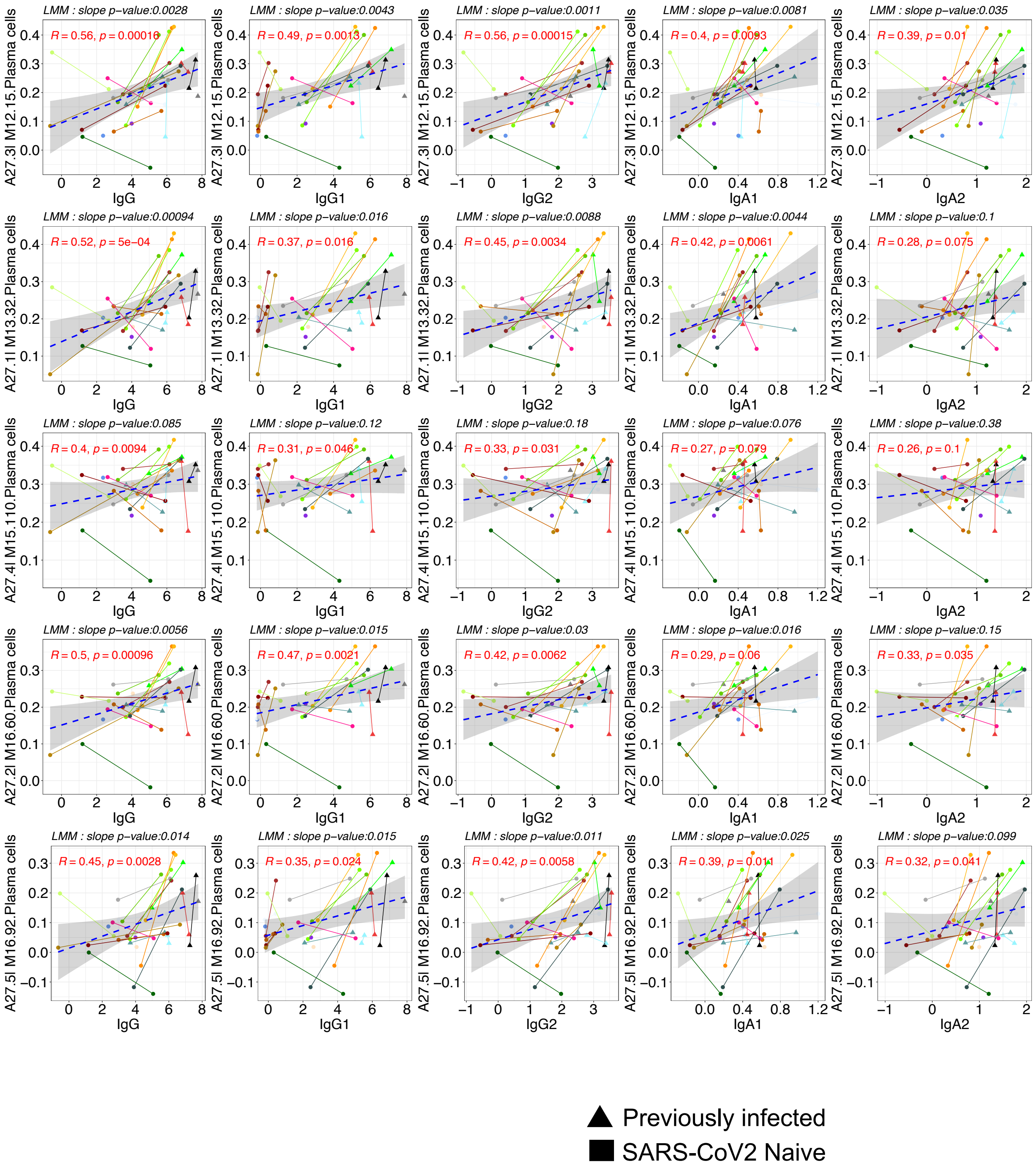


**Figure S12: Association of SARS-CoV2 S1 specific antibody responses measured on day 14 post second-dose with A27/plasmablast response measured on day 4 post-second dose:** The correlation plots indicate the degree of association between interferon module scores (Y-axis: computed as single sample gene enrichment score) and antibody index (X-Axis). Spearman's correlation R value and p-value are shown on each of the plots, along with slope p-value (from linear mixed-effect modeling - see methods for details). One baseline pre-vaccine and one post-

vaccine time point are shown for each individual subject (color-coded by donor and connected by a line. In addition: squares = SARS-CoV2 Naïve, triangles = previously infected and recovered). Results are shown for 5 different A27/plasmablast modules (rows) across multiple antibody isotypes (columns).

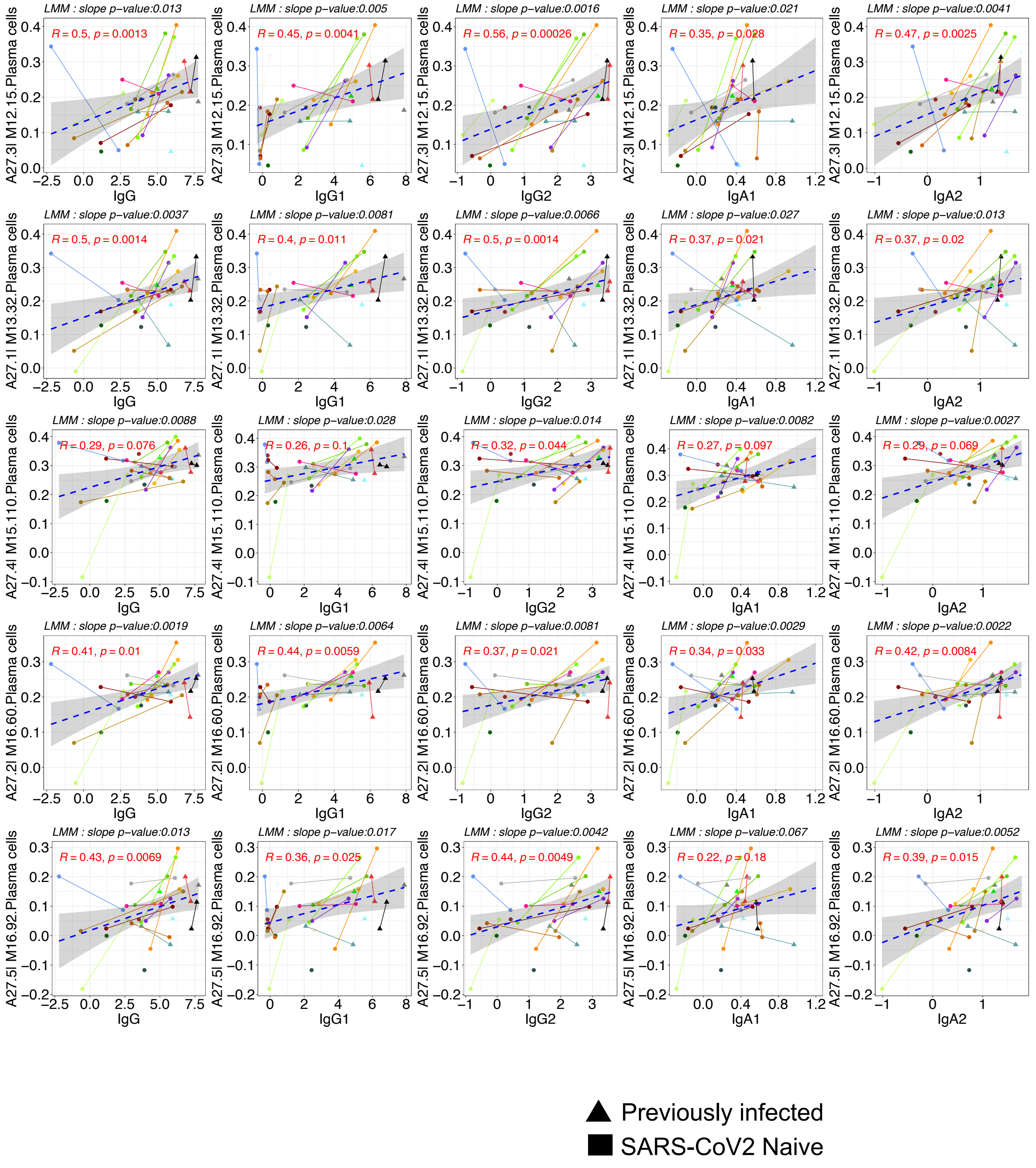


**Figure S13: Association of SARS-CoV2 S1 specific antibody responses measured on day 14 post second-dose with A27/plasmablast response measured on day 5 post-second dose:** The correlation plots indicate the degree of association between interferon module scores (Y-axis:

computed as single sample gene enrichment score) and antibody index (X-Axis). Spearman's correlation R value and p-value are shown on each of the plots, along with slope p-value (from linear mixed-effect modeling - see methods for details). One baseline pre-vaccine and one post-vaccine time point are shown for each individual subject (color-coded by donor and connected by a line. In addition: squares = SARS-CoV2 Naïve, triangles = previously infected and recovered). Results are shown for 5 different A27/plasmablast modules (rows) across multiple antibody isotypes (columns).

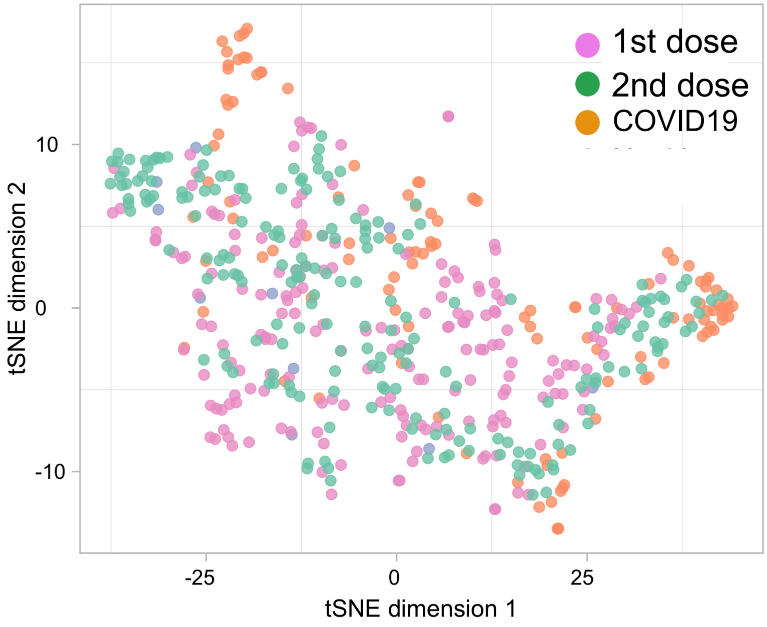


**Figure S14: Clustering of vaccination and COVID-19 studies samples according to patterns of interferon responses colored according to study group.** Similarities in patterns of interferon response induction across the six modules forming aggregate A28 among samples from our vaccination cohort and one of our COVID-19 disease cohort (PREDICT-19 / Italy) are represented on a tSNE plot. Samples are color coded according to study groups: COVID-19 samples are shown in orange along with specific post-vaccination timepoints (pink: post-first dose days 1 and 2, green: post-second dose days 1 and 2).

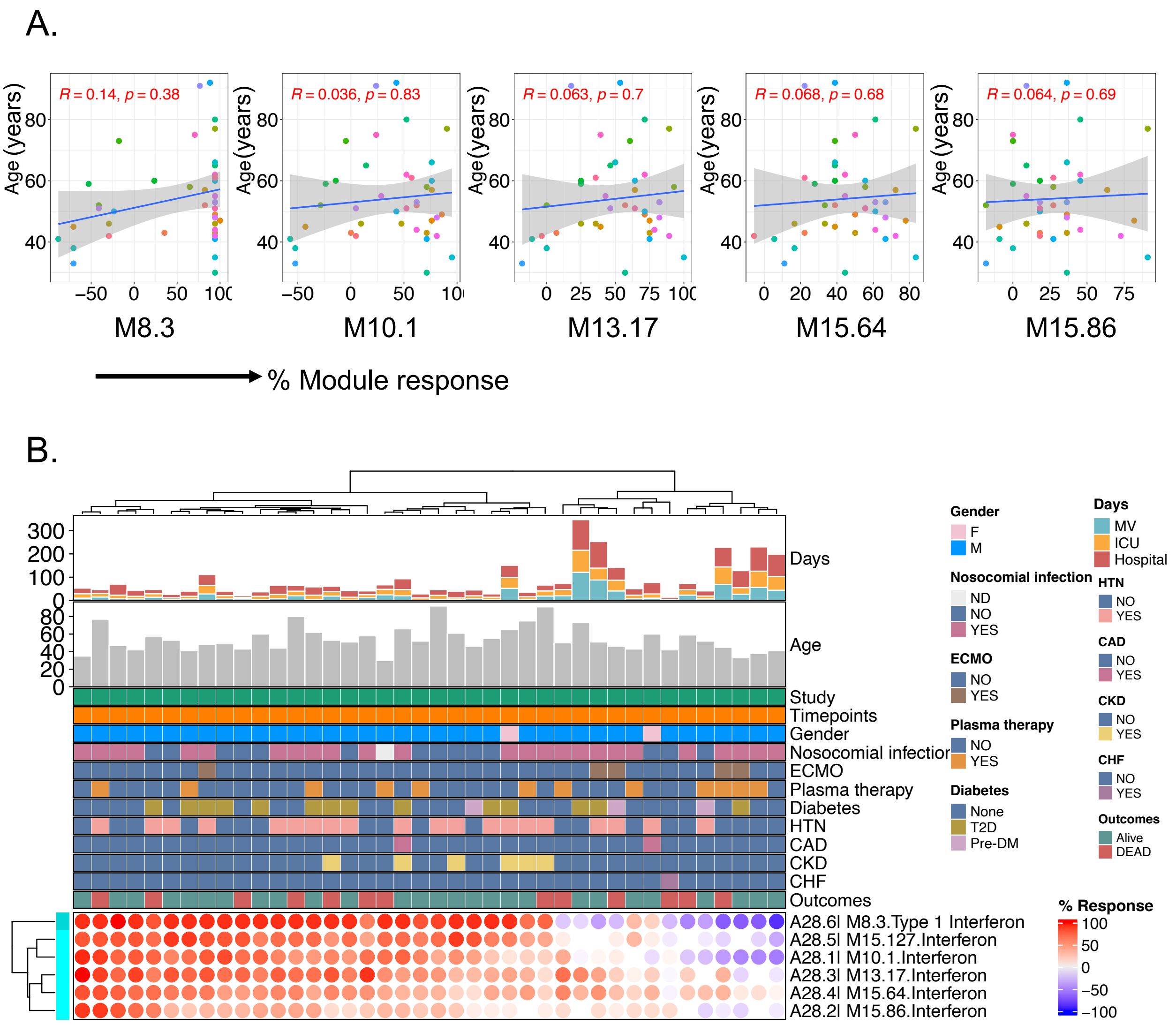


**Figure S15: Interferon phenotypes and length of ICU stay is not associated with age. (A)** The correlation plots indicate the degree of association between age (Y-axis) and module response (X-Axis: percentage of genes constituting a given module showing increase in abundance over pre-vaccination baseline). Spearman's correlation was used, and R values and p-values are shown on each of the plots. Each point represents an individual subject. Results are shown for 6 different interferon response modules **(B)** The fingerprint heatmap shows patterns of interferon responses for COVID-19 patients with severe disease upon ICU admission. The top-most panel (bar graph) shows length of stay in the ICU, the hospital or on mechanical ventilation (MV). The panel below shows age. The colored tracks show different clinical parameters (ECMO [Extracorporeal Membrane Oxygenation], HTN [Hypertension], CAD [Coronary Artery Disease], CKD [Chronic Kidney Disease], CHF [Congestive Heart Failure]).

## Membership of the PREDICT-19 consortium:

| name | affiliation |
| --- | --- |
| Amy L Phu | Department of Intensive Care Medicine, Nepean Hospital, NSW, Australia Westmead Clinical School, Sydney Medical School, University of Sydney, Sydney, NSW, Australia |
| Ya Wang | 1. Sydney Medical School, Nepean Hospital, Sydney, Australia 2. Centre for Immunology and Allergy Research,Westmead Institute for Medical Research, Sydney, Australia 3.Department of Intensive Care Medicine, Nepean Hospital, Sydney, Australia |
| Stephen Macdonald | 1. Centre for Clinical Research in Emergency Medicine, Harry Perkins Institute of Medical Research 2. Royal Perth Hospital, University of Western Australia, Perth, WA, Australia |
| Tracy Chew | Sydney Informatics Hub, Core Research Facilities, University of Sydney |
| Nandan Deshpande | Sydney Informatics Hub, Core Research Facilities, University of Sydney |
| Gunawan Gunawan | Medistra Hospital, Jakarta Indonesia |
| Jennifer Audsley | Department of Infectious Diseases, University of Melbourne, at the Peter Doherty Institute for Infection and Immunity, Melbourne, Victoria, Australia |
| Marek Nalos | Australian National University, Goulburn Clinical School NSW, Australia, Charles University Prague Medical Faculty PLZEŇ Czechia |
| Timothy Kwan | University of Sydney, Sydney |
| Mohammed Toufiq | Research Branch, Sidra Medicine |
| Karan Kim | The Westmead Institute for Medical Research |
| Alberto Ballestrero | DIMI Università di Genova and IRCCS Ospedale Policlinico San Martino |
| Rostyslav Bilyy | Danylo Halytsky Lviv National Medical University, 79010, Lviv, Ukraine |
| Win Sen Kuan | Emergency Medicine Department, National University Hospital, Singapore; Department of Surgery, Yong Loo Lin School of Medicine, National University of Singapore, Singapore |
| Klaus Schughart | University of Tennessee Health Science Center, Dept. of Microbiology Immunology and Biochemistry, Memphis, U.S.A. |
| Marko Radic | Department of Microbiology, Immunology and Biochemistry; University of Tennessee Health Science Center, Memphis, TN (USA) |
| Tiana Pelaia | Department of Intensive Care Medicine, Nepean Hospital, NSW, Australia |
| Philip N Britton | Sydney Medical School, University of Sydney |
| Sally Teoh | Department of Intensive Care Medicine, Nepean Hospital, Sydney, Australia |
| Martin Matejovic | 1st Department of Internal Medicine, Faculty of Medicine in Pilsen, Pilsen University Hospital, Charles University Prague, Alej Svobody 80, 323 00 Pilsen Czech Republic |
| Narasaraju Teluguakula | Department of Microbiology, Adichunchanagiri Institute of Medical Sciences, Adichunchanagiri University BG Nagara, Karnatka, India |
| Rodolphe Thiebaut | Bordeaux University, France |
| Kirsty R. Short | 1 School of Chemistry and Molecular Biosciences, The University of Queensland, Brisbane, Queensland, Australia 2 Australian Infectious Diseases Research Centre, Global Virus Network Centre of Excellence, Brisbane, Queensland, Australia |
| Michele Bedognetti | ASL 3 Genovese, Genoa, Italy |
| Arutha Kulasinghe | The University of Queensland Diamantina Institute, The University of Queensland |

| | |
|---|---|
| Tri Giang PHAN | 1 Garvan Institute of Medical Research, Sydney, Australia; 2 St Vincent's Healthcare Clinical Campus, School of Clinical Medicine, Faculty of Medicine and Health, UNSW Sydney, Sydney, Australia |
| Carl G Feng | 1.Guangdong Key Laboratory of Regional Immunity and Diseases, Department of Pathogen Biology, Shenzhen University School of Medicine, Shenzhen, China 2. Immunology and Host Defense Group, School of Medical Sciences, Faculty of Medicine and Health, the University of Sydney, Sydney, Australia |
| Yoann Zerbib | Intensive Care Unit, Amiens Picardie University Hospital, Amiens, France |
| Nicholas West | Systems Biology and Data Science and Mucosal Immunology Research Group, Menzies Health Institute Queensland, Griffith University, Queensland, Australia |
| Thomas Karvunidis | Medical ICU, 1st. Dept. of Internal Medicine, Charles University, Faculty of Medicine, Teaching Hospital and Biomedical Center in Pilsen, Alej Svobody 80, 323 00, Pilsen, Czech Republic |
| Marcela Kralovcova | Medical ICU, 1st. Dept. of Internal Medicine, Charles University, Faculty of Medicine, Teaching Hospital and Biomedical Center in Pilsen, Alej Svobody 80, 323 00, Pilsen, Czech Republic |
| Adrea, De Maria | 1.Department of Health Sciences, Unifersity of Genoa, Italy 2.Division of infectious diseases, Policlinico Ospedale San Martino, IRCCS, Genova, Italy |
| Maryam Shojaei | 1. Sydney Medical School, Nepean Hospital, Sydney, Australia 2. Centre for Immunology and Allergy Research,Westmead Institute for Medical Research, Sydney, Australia 3.Department of Intensive Care Medicine, Nepean Hospital, Sydney, Australia |
| Velma Herwanto | 1. Faculty of Medicine, Universitas Tarumanagara, Jakarta, Indonesia 2. Department of Internal Medicine, Siloam Hospitals Kebon Jeruk, Jakarta, Indonesia |
| Irani Thevarajan | Doherty Institute for Infection and Immunity The Royal Melbourne Hospital |
| Ricardo Garcia Branco | Sidra Medicine, Doha, Qatar |
| Anthony McLean | 1. Sydney Medical School, Nepean Hospital, Sydney, Australia 2.Department of Intensive Care Medicine, Nepean Hospital, Sydney, Australia |
| Ben Tang | 1.Centre for Immunology and Allergy Research,Westmead Institute for Medical Research, Sydney, Australia 2.Department of Intensive Care Medicine, Nepean Hospital, Sydney, Australia |
| Jonathan Iredell | 1. Centre for Infectious Diseases and Microbiology, Westmead Institute for Medical Research, Sydney, Australia 2. Faculty of Medicine and Health, School of Medical Sciences, University of Sydney, Sydney, Australia 3. Westmead Hospital, Western Sydney Local Healthy District, Sydney, Australia |
| John- Sebastian Eden | Westmead Institute for Medical Research, Sydney, Australia |
| Alan Cripps | Griffith University Queensland, Australia |
| Davide Bedognetti | Sidra Medical and Research Center, Doha, Qatar |
| Gabriele Zoppoli | 1.Department of Internal Medicine, DiMI, University of Genova, Italy 2. Ospedale Policlinico San Martino, Genova, Italy |
| Miroslav Prucha | Dept. of Clinical Biochemistry, Haematology and Immunology, Na Homolce Hospital,, Roentgenova 2, Prague 5, Czech Republic |
| Paolo Cremonesi | 1. Dipartimento di Emergenza ed Accettazione (D.E.A.)2. Struttura Complessa Medicina e Chirurgia D' Accettazione e D' Urgenza E.O. Ospedali Galliera Genova Mura delle Cappuccine 14 16128 Genova |

| | |
|---|---|
| Darawan Rinchai | The Rockefeller University, Laboratory of Human Genetics of Infectious Diseases, New York, NY, USA |
| Damien Chaussabel | The Jackson Laboratory, Computational Sciences Department, Farmington, CT, USA |


**List of supplementary data files**:

**Supplementary File 1:** A file in the MS Excel .xlsx format including study subject serological profiles to a stabilized trimer of Spike protein, its receptor-binding domain, the Nucleo and Envelope proteins of SARS-CoV2, and the subunit S1 of the SARS Spike protein.

**Supplementary File 2:** A file in the MS Excel .xlsx format including results from the differential gene-set enrichment analysis run on post-first vaccine dose transcriptome profiling data. This analysis determined the significance of temporal changes of the 382 modules constituting the BloodGen3 repertoire.

**Supplementary File 3:** A file in the MS Excel .xlsx format including the gene composition and the functional annotations of the six A28 interferon-response modules that belong to the BloodGen3 repertoire.

**Supplementary File 4:** A file in the MS Excel .xlsx format including results from the differential gene-set enrichment analysis run on post-second vaccine dose transcriptome profiling data. This analysis determined the significance of temporal changes of the 382 modules constituting the BloodGen3 repertoire.
